# Defects in leaf carbohydrate metabolism compromise acclimation to high light and lead to a high chlorophyll fluorescence phenotype in *Arabidopsis thaliana*

**DOI:** 10.1186/1471-2229-12-8

**Published:** 2012-01-16

**Authors:** Jessica Schmitz, Mark Aurel Schöttler, Stephan Krueger, Stefan Geimer, Anja Schneider, Tatjana Kleine, Dario Leister, Kirsten Bell, Ulf-Ingo Flügge, Rainer E Häusler

**Affiliations:** 1 University of Cologne, Botanical Institute, Biocenter Cologne, Zülpicher Str. 47B, D-50674 Cologne, Germany; 2 Max Planck Institute of Molecular Plant Physiology, Am Mühlenberg 1, D-14476 Potsdam-Golm, Germany; 3 Universität Bayreuth, Zellbiologie/Elektronenmikroskopie NW I/B1, Universitätsstrasse 30, D-95447 Bayreuth, Germany; 4 Biozentrum der Ludwig-Maximilians-Universität München, Department Biologie I - Botanik Großhaderner Str. 2-4, D-82152 Planegg-Martinsried, Germany

## Abstract

**Background:**

We have studied the impact of carbohydrate-starvation on the acclimation response to high light using *Arabidopsis thaliana *double mutants strongly impaired in the day- and night path of photoassimilate export from the chloroplast. A complete knock-out mutant of the triose phosphate/phosphate translocator (TPT; *tpt-2 *mutant) was crossed to mutants defective in (i) starch biosynthesis (*adg1-1, pgm1 *and *pgi1-1; *knock-outs of ADP-glucose pyrophosphorylase, plastidial phosphoglucomutase and phosphoglucose isomerase) or (ii) starch mobilization (*sex1-3*, knock-out of glucan water dikinase) as well as in (iii) maltose export from the chloroplast (*mex1-2*).

**Results:**

All double mutants were viable and indistinguishable from the wild type when grown under low light conditions, but - except for *sex1-3/tpt-2 *- developed a high chlorophyll fluorescence (HCF) phenotype and growth retardation when grown in high light. Immunoblots of thylakoid proteins, Blue-Native gel electrophoresis and chlorophyll fluorescence emission analyses at 77 Kelvin with the *adg1-1/tpt-2 *double mutant revealed that HCF was linked to a specific decrease in plastome-encoded core proteins of both photosystems (with the exception of the PSII component cytochrome b_559_), whereas nuclear-encoded antennae (LHCs) accumulated normally, but were predominantly not attached to their photosystems. Uncoupled antennae are the major cause for HCF of dark-adapted plants. Feeding of sucrose or glucose to high light-grown *adg1-1/tpt-2 *plants rescued the HCF- and growth phenotypes. Elevated sugar levels induce the expression of the glucose-6-phosphate/phosphate translocator2 (GPT2), which in principle could compensate for the deficiency in the TPT. A triple mutant with an additional defect in GPT2 (*adg1-1/tpt-2/gpt2-1*) exhibited an identical rescue of the HCF- and growth phenotype in response to sugar feeding as the *adg1-1/tpt-2 *double mutant, indicating that this rescue is independent from the sugar-triggered induction of GPT2.

**Conclusions:**

We propose that cytosolic carbohydrate availability modulates acclimation to high light in *A. thaliana*. It is conceivable that the strong relationship between the chloroplast and nucleus with respect to a co-ordinated expression of photosynthesis genes is modified in carbohydrate-starved plants. Hence carbohydrates may be considered as a novel component involved in chloroplast-to-nucleus retrograde signaling, an aspect that will be addressed in future studies.

## Background

The majority of the CO_2 _assimilated in the Calvin-Benson cycle is eventually converted into carbohydrates. These can be retained inside the chloroplast in form of transitory starch or exported from the chloroplast and further transported via the phloem in form of sucrose to supply sink tissues such as roots, flowers and developing seeds with carbon and energy. The triose phosphate/phosphate translocator (TPT) of the inner envelope membrane of chloroplasts is the major interface for the day path of photoassimilate export from the stroma [1]. Triose phosphates exported in the light are used as precursors for sucrose biosynthesis in the cytosol. In the night path of photoassimilate export, transitory starch is degraded via β-amylase, isoamylase and disproportionating enzyme (DPE1) yielding maltose and glucose as end products [2,3]. The transporters responsible for the export of glucose and maltose have been identified and the respective mutants characterized [4-7]. In the cytosol, maltose is further metabolized via DPE2 [8,9], a cytosolic heteroglycan [10] and cytosolic glucan phosphorylase (in *A. thaliana *PSH2) resulting in glucose and glucose-1-phosphate (Glc1P), which enter further metabolism [11].

Surprisingly, an *A. thaliana *knock-down TPT mutant (*tpt-1; *identical to *ape2*, [*acclimation of photosynthesis to environment2*; [12]) lacked any pronounced development or growth phenotype [13], mainly because the reduced TPT transport capacity can be compensated for by an increased starch turnover in the light [13,14] like previously reported for antisense TPT tobacco plants [15]. Hence, sucrose biosynthesis can be maintained in the light by the night path of photoassimilate export, i.e. starting from maltose and glucose. Strikingly, crosses of *tpt-1 *with *adg1-1 *[16] or *sex1-1 *[17], defective in the catalytic subunit of ADPglucose phosphorylase (AGPase) or glucan water dikinase (GWD, [18]), were still viable albeit retarded in growth, suggesting that the residual TPT activity permits survival of the plants [13]. In particular, the *adg1-1/tpt-1 *double mutant exhibited a severe growth and photosynthesis phenotype reminiscent of potato plants with an antisense inhibition of both the TPT and AGPase [19]. The *A. thaliana *double mutant is characterized by impaired photosynthetic electron transport, changes in the chloroplast ultrastructure, such as grana hyperstacking, and increased numbers of plastoglobules, high Chl fluorescence (HCF) in the dark-adapted state, and a perturbed redox equilibrium in the light and dark [20]. It has been proposed that residual TPT activity in the *tpt-1 *allele permits survival of the *adg1-1/tpt-1 *double mutant [20].

A full knock-out mutant of the *TPT *gene (i.e. *tpt-2*) has been isolated and crossed not only to *adg1-1*, but also to further mutants defective in starch biosynthesis, its mobilization or the export of the starch degradation product maltose from the chloroplasts. All double mutants were viable and exhibited a pronounced growth and/or photosynthesis phenotype only when grown under high light, but not under low light conditions.

Here we have focused on the mechanistic basis of the HCF phenotype and the role of soluble sugars in the acclimation to high light intensities in mutants compromised in carbohydrate metabolism. The HCF phenotype of the *adg1-1/tpt-1 *double mutant allele could only be partially explained by an increased rate of chlororespiration and the concomitant enhanced reduction state of the plastoquinone pool in the dark. This notion was mainly based on the additional increase in the ground Chl-*a *fluorescence (F_o_) in the absence of O_2 _or following the application of octyl gallate, an inhibitor of the plastid localized alternative oxidase, a central component involved in chlororespiration [20]. The mechanistic basis for the HCF phenotype has now been resolved. It is mainly caused by light harvesting complexes (LHCs) uncoupled from their reaction centers.

The roles of soluble sugars and the glucose-6-phosphate/phosphate translocator2 (GPT2) in the altered acclimation response of *adg1-1/tpt-2 *to high light has been addressed by feeding sucrose or glucose to the double mutant or an *adg1-1/tpt-2/gpt2-1 *triple mutant. GPT2 has been shown to be strongly induced both at a transcriptional and functional level in leaves of mutants incapable of sufficient starch biosynthesis, such as *adg1-1, pgm1 *or *pgi1-2 *as well as in *adg1-1/tpt-1 *[21]. The induction of GPT2 correlates with the accumulation of soluble sugars in the mutant's background. Our data demonstrate that soluble sugars are key players in the compromised or modified acclimation response to high light in *adg1-1/tpt-2 *and that GPT2 is not involved in this response.

## Results

### Isolation of a knock-out mutant of the *TPT *gene (*tpt-2*) and generation of double mutants

**Figure 1:**
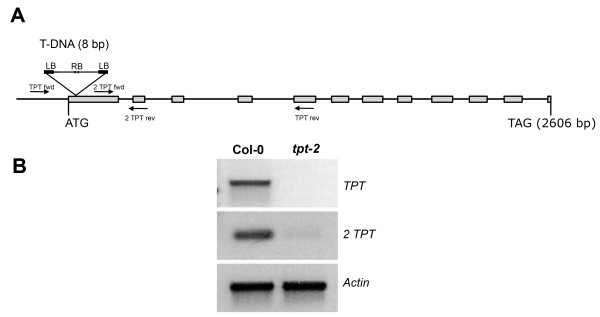
**Molecular characterization of the *tpt-2 *T-DNA insertion mutant**. (A) Position of the T-DNA insertion in the TPT gene as well as primers used for RT-PCR. (B) Transcript levels of the *TPT *as well as the *actin *control in wild-type and *tpt-2 *plants determined by RT-PCR using the primer pairs TPT fwd and TPT rev (*TPT*) or 2 TPT fwd and 2 TPT rev (*2 TPT*). The low amount of the amplified *2 TPT *product is due to a second ATG 32 bp downstream of the start ATG. However, this ATG is not within the reading frame

The *tpt-1 *mutant (WS-2 background), carrying a T-DNA insertion in the promoter region 24 bp upstream of the start ATG, showed residual transcripts of the *TPT *gene and substantial TPT transport activities of up to 16% of the wild type [13,21]. In order to study the consequences of a complete knock-out of the *TPT *gene in *A. thaliana*, we isolated the *tpt-2 *allele (Col-0 background; N573707; SALK_073707.54.25.x), which carries a T-DNA insertion 8 bp downstream of the start ATG (Figure 1A). The *tpt-2 *mutant plants completely lacked *TPT *specific transcripts (Figure 1B), and P_i _transport decreased by 44% from 2.94 ± 0.35 nmol·g^-1 ^fw in the wild type to 1.64 ± 0.02 nmol·g^-1 ^fw in *tpt-2*. Transport of 3-PGA (3.07 ± 0.41 nmol·g^-1 ^fw in wild-type plants) was not detectable in *tpt-2*, demonstrating that the *tpt-2 *allele represents a loss-of-function mutant of the TPT (the above data express the mean ± SE of n = 4 experiments). In order to simultaneously block the day- and night path of carbon export from the chloroplasts, the *tpt-2 *mutant has been crossed to the starch-free or low-starch mutants *adg1-1 *[16], *pgm1 *[22] and *pgi1-1 *[23] as well as to mutants compromised in starch mobilization (*sex1-3*, [24]) or export of maltose from the chloroplast (*mex1-2*, [5]). Despite the complete loss of TPT activity all double mutants are viable.

### The phenotypes of *adg1-1/tpt-2 *emerged only under high light conditions

**Figure 2:**
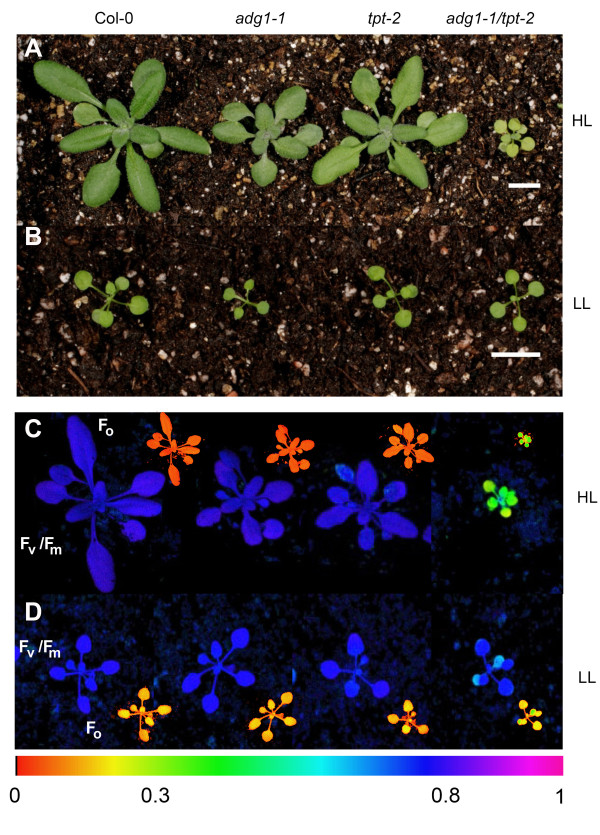
**Phenotypes and Chl-*a *fluorescence images of wild-type and mutant plants**. Phenotypic appearance of Col-0, *adg1-1, tpt-2 *and *adg1-1/tpt-2 *grown in HL (A) or LL (B) for 4 weeks as well as Chl-*a *fluorescence false color images of the F_v_/F_m _ratio of the same lines grown in HL (C) or LL (D). The smaller insets in (C) and (D) show images of F_o_. Care was taken that the distance between the leaf surface of the plants and the light source was constant in all experiments. The color scale indicates the numeric values of F_v_/F_m _ratios or of F_o _(in a V-scale). The size bars in (A) and (B) indicate 1 cm

Like *adg1-1/tpt-1 *the *adg1-1/tpt-2 *double mutant allele is retarded in vegetative growth and shows a HCF phenotype in the dark-adapted state [20]. Both phenotypes emerged when double mutant plants were grown at a photosynthetic photon flux density (PDF) well above 50-70 μmol·m^-2^·s^-1^. However, at a PFD below 50 μmol·m^-2^·s^-1^, *adg1-1/tpt-2 *lacked any prominent phenotype. An impaired day and night path of carbon export from the chloroplast compromises the redox equilibrium in the stroma and photosynthesis. Both are strongly dependent on the light intensity the plants experience during growth. Here we compared the characteristics of mutant and wild-type plants grown under low light (LL) conditions (i.e. a PFD of 30 μmol·m^-2^·s^-1^) with those grown at a ten-fold higher PFD (i.e. 300 μmol·m^-2^·s^-1^), defined as high light (HL) conditions. All plant lines were considerably smaller when grown in LL (Figure 2B) compared to HL (Figure 2A) and *adg1-1/tpt-2 *grown in LL lacked any pronounced growth- or HCF phenotype (Figure 2B and 2D; Table 1A). However, the characteristic phenotypes of the double mutant were well marked in HL-grown plants (Figure 2A and 2C). Vegetative growth was substantially diminished (Figure 2A) and dark-adapted *adg1-1/tpt-2 *plants exhibited a characteristic increase in the ground fluorescence (F_o_), which was reflected in a decrease in the F_v_/F_m _ratio from 0.8 in wild-type or single mutant to around 0.3 in double mutant plants (Figure 2C, Table 1B). In contrast, LL-grown *adg1-1/tpt-2 *plants failed to exhibit a HCF phenotype during their development. In parallel with growth retardation and the HCF phenotype Chl-, carotenoid- and protein contents were diminished only in HL-grown double mutant compared to wild-type and single mutant plants (Table 1B). The Chl *a/b *ratio was decreased from around 3 in the wild type and the single mutants to 2.1 in HL-grown double mutants and was also slightly lower in LL-grown *adg1-1/tpt-2 *plants (Table 1A).

**Table 1:** Chl-*a *fluorescence parameters as well as pigment and protein contents of the *adg1-1/tpt-2 *double mutant compared to the wild type (Col-0) and the *adg1-1 *and *tpt-1 *single mutants

Lines	**F**_**v**_**/F**_**m **_**ratio**	ΦPSII	Chl content (mg·m^-2^)	Chl *a/b *ratio	Carotenoid content (mg·m^-2^)	Protein content (g·m^-2^)
A Plants grown in LL						

Col-0	0.744 ± 0.011	0.584 ± 0.016 ^**b, d**^	127.2 ± 7.5	2.68 ± 0.03 ^**d**^	4.4 ± 0.3	0.59 ± 0.10

*adg1-1*	0.751 ± 0.010	0.487 ± 0.077 ^**a, c**^	133.8 ± 16.6	2.64 ± 0.06 ^**d**^	4.5 ± 0.6	0.57 ± 0.14

*tpt-2*	0.753 ± 0.019	0.582 ± 0.030 ^**b, d**^	135.8 ± 11.3	2.61 ± 0.08 ^**d**^	4.5 ± 0.3	0.44 ± 0.04

*adg1-1/tpt-2*	0.746 ± 0.005	0.472 ± 0.022 ^**a, c**^	135.3 ± 9.2	2.35 ± 0.07 ^**a, b, c**^	4.7 ± 0.4	0.54 ± 0.05

B Plants grown in HL						

Col-0	0.800 ± 0.007 ^**c, d**^	0.396 ± 0.014 ^**d**^	286.2 ± 26.5 ^**b, c, d**^	3.09 ± 0.13 ^**d**^	10.6 ± 0.5 ^**b, d**^	3.38 ± 0.42 ^**b, d**^

*adg1-1*	0.778 ± 0.007 ^**d**^	0.366 ± 0.014 ^**d**^	320.6 ± 24.3^** a, c, d**^	3.00 ± 0.04 ^**c, d**^	12.4 ± 0.9 ^**a, c, d**^	4.29 ± 0.64 ^**a, c, d**^

*tpt-2*	0.757 ± 0.030 ^**a, d**^	0.359 ± 0.046 ^**d**^	258.7 ± 9.9^** a, b, d**^	3.11 ± 0.07 ^**b, d**^	10.1 ± 0.6 ^**b, d**^	3.24 ± 0.42 ^**b, d**^

*adg1-1/tpt-2*	0.226 ± 0.021 ^**a, b, c**^	0.056 ± 0.027 ^**a, b, c**^	103.0 ± 10.8^** a, b, c**^	2.13 ± 0.04 ^**a, b, c**^	5.5 ± 0.5 ^**a, b, c**^	2.26 ± 0.48 ^**a, b, c**^

The plants were grown for 4 weeks either in LL (A) or HL (B). Photosynthesis parameters were determined with the Imaging-PAM fluorometer using the integrated light source. Before the determination of F_v_/F_m _ratios the plants were dark-adapted for 15 min. The ΦPSII values refer to steady state photosynthesis, which was attained 7-10 min after onset of illumination with PDFs similar to those the plants experienced during growth (i.e. at 30 μmolm^-2^·s^-1 ^[LL] and 300 μmol·m ^-2^·s ^-1 ^[HL], respectively). The data represent the mean ± SE of n = 3 (F_v_/F_m _ratio, ΦPSII) or n = 5 (Chl content, Chl *a/b *ratio, carotenoid- and protein content) measurements. Significant differences in the data pairs were identified by the ANOVA test and further analyzed by the Tukey-Kramer test. The letters as superscripts denote the lines to which a significant difference (*P *< 0.05) was found with a = Col-0, b = *adg1-1*, c = *tpt-2 *and d = *adg1-1/tpt-2*.

**Figure 3:**
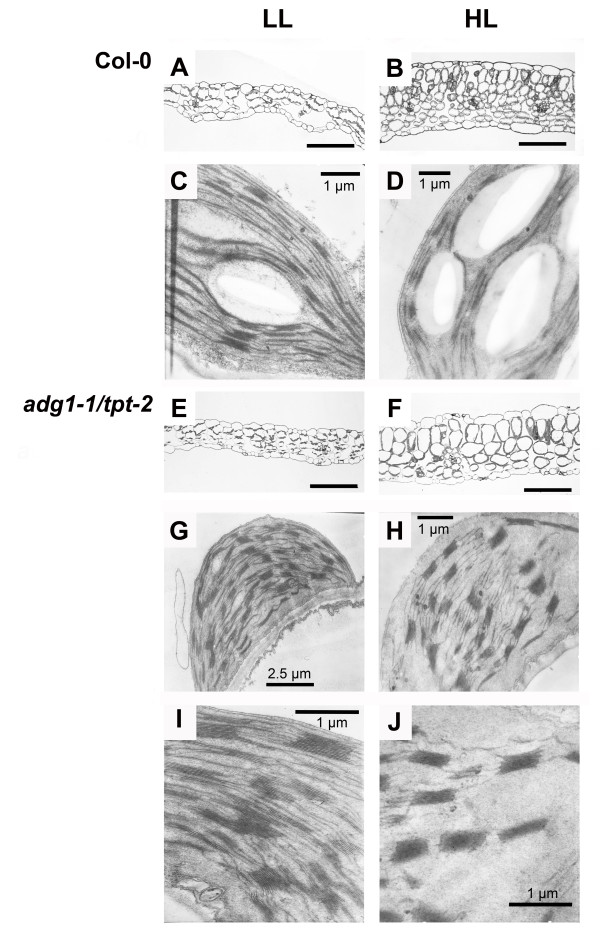
**Structural analyses of leaf cross sections or chloroplast of LL- and HL-grown wild-type and *adg1-1/tpt-2 *plants**. Leaf cross sections (A, B, E, F) and chloroplast ultrastructure (C, D, G-I) obtained by light microscopy or TEM of wild-type (A-D) or *adg1-1/tpt-2 *double mutant plants (E-J) grown in LL (A, C, E, G, I) or HL (B, D, F, H, J). The bars in (A, B, E, F) represent 100 μm

The growth and photosynthesis phenotypes of *adg1-1/tpt-2 *were accompanied by an altered leaf anatomy and chloroplast ultrastructure compared to the wild type (Figure 3). In general, leaves of HL-grown plants were thicker (Figure 3B and 3F) compared to those grown in LL (Figure 3A and 3E). However, compared to the wild type, HL-grown *adg1-1/tpt-2 *contained less, but larger mesophyll cells (Figure 3B and 3F).

With regard to the chloroplast ultrastructure, the wild type exhibited a higher number of grana stacks when grown under LL-conditions (Figure 3C, on average 5.1 stacks per granum) compared to HL-conditions (Figure 3D; on average 3.9 stacks per granum). Strikingly, grana stacking in chloroplasts of *adg1-1/tpt-2 *was even increased from 6.7 to 8.3 stacks per granum in LL- compared to HL-grown plants (Figure 3G to 3J). Additional File 1 shows a comparison of the distribution of 'grana stack number classes' between LL- and HL- grown wild-type and double mutant plants. Under both growth light conditions *adg1-1/tpt-2 *showed a much broader distribution of 'grana stack number classes' with up to 17 stacks per granum in HL-grown plants compared to the wild type with a maximum of 9 stacks per granum.

Taken together, the above data indicate that the phenotypic changes in *adg1-1/tpt-2 *are light dependent and suggest that mechanisms leading to a proper acclimation to HL are compromised in the double mutant compared to wild-type or single mutant plants.

### Composition and abundance of photosynthetic multiprotein complexes and supercomplexes are strongly altered in HL-grown *adg1-1/tpt-2 *plants

**Figure 4:**
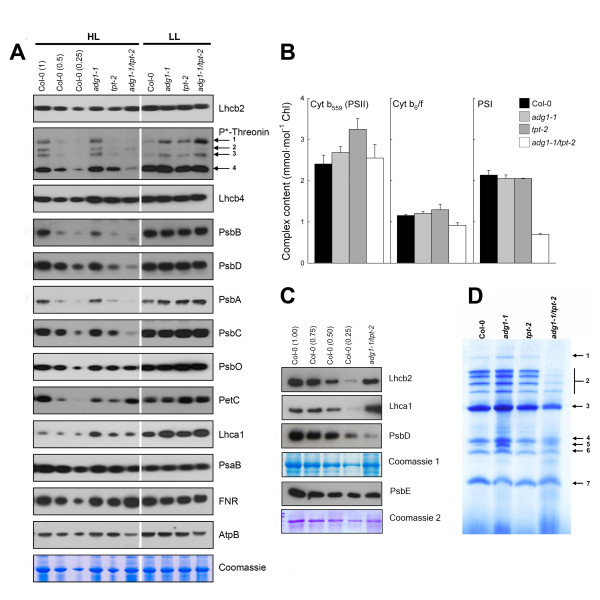
**Immunoblots of thylakoid proteins, spectroscopic determinations of functional PS components, and separation of PS complexes by Blue-Native gel electrophoresis**. (A) Immunoblots of thylakoid proteins associated with photosynthesis after separation of total proteins (approximately 10 μg per lane) isolated from leaves of HL- and LL- grown Col-0 wild type, the *adg1-1 *and *tpt-2 *single mutants as well as the *adg1-1/tpt-2 *double mutant on SDS-PAGE. Note that the Coomassie staining for proteins of HL-grown *tpt-2 *corresponds to about 0.5-fold rather than 1-fold of Col-0 protein. P*-Threonin indicates signals obtained following incubation of the blots with a phospho-threonin antibody. The numbers indicate signals from PsbC (1), CaS (2), PsbA/PsbD (3), and LhcbII (4). (B) Spectroscopic determinations of functional components of PSII, PSI and the cyt b_6_/f complex. (C) Immunoblots of PsbE in comparison to Lhcb2, Lhca1, and PsbE in HL-grown wild-type and *adg1-1/tpt-2 *plants. (D) Separation of thylakoid proteins of HL-grown Col-0 wild type, the *adg1-1 *and *tpt-2 *single mutants as well as the *adg1-1/tpt-2 *double mutant on Blue-Native gels. The numbers indicate individual protein fractions of the isolated thylakoids such as the NDH complex (1, see Additional file 2: Table S2), PSII super complexes (2), PSI supercomplex/PSII dimer (3), ATPase (4), PSII monomers (5), PSII/PsbC (6), and LHCII trimers (7)

Taking the HCF phenotype and increased grana stacking in both the *adg1-1/tpt-1 *and *adg1-1/tpt-2 *alleles into account, we investigated the accumulation of photosynthesis-related proteins and corresponding multiportein complexes by immunoblot analysis (Figure 4A and 4C) and by Blue-Native PAGE (Figure 4D). HL-grown *adg1-1/tpt-2 *plants exhibited substantial changes in the abundance of PSII core components compared to the wild type and the single mutants. The plastome-encoded subunits PsbB (CP47), PsbD (D2), PsbA (D1), and PsbC (CP43) were hardly detectable in the double mutant, whereas the abundance of PSII-associated Lhcb proteins, as well as the oxygen evolving complex (OEC; PsbO), which are nuclear-encoded proteins, remained unaffected. In contrast, when plants were grown in LL, the PSII core protein contents in *adg1-1/tpt-2*, wild-type and single mutant plants were very similar (Figure 4A). The Cyt b_6_/f complex component PetC (Rieske protein) was decreased in the HL-grown double mutant as well as in the single mutants compared to the wild type (Figure 4A). The plastome-encoded PSI core component PsaB (P700 apoprotein A2), which together with PsaA (P700 apoprotein A1) is involved in electron transfer from plastocyanin to the PSI acceptor side [25], was diminished by 50-70% in HL-grown *adg1-1/tpt-2*, but remained unchanged in the single mutants irrespectively of the growth PDF. Nuclear-encoded Lhca1 was increased in all HL-grown mutant lines compared to the wild type (Figure 4A). Moreover, ferredoxin:NADP reductase (FNR), which is also nuclear-encoded, was increased in the double mutant grown under HL-condition (Figure 4A), whereas, under HL- and LL-conditions, the abundance of the plastome-encoded ATPase subunit AtpB was slightly decreased in *adg1-1/tpt-2*.

Furthermore, the phosphorylation state of several thylakoid proteins was analyzed. Phosphorylation levels of Lhcb2, CP43, CaS (a calcium sensing receptor residing in the stroma thylakoids; [26]), and D1/D2 were substantially diminished in HL-grown *adg1-1/tpt-2 *compared to wild-type and single mutant plants (Figure 4A). The decrease in the D1/D2 phosphorylation state correlates well with its overall diminished protein content, whereas the phosphorylation state of Lhcb2 was decreased despite unaffected protein levels (Figure 4A).

Spectroscopic analyses of functional components of the photosynthetic electron transport chain in isolated thylakoids supported the data obtained by immunoblots (Figure 4B). PSI contents were diminished by more than 50% only in HL-grown double mutants, but not in the single mutants compared to the wild type. These data are well in line with immunoblots against the PSI core component PsaB (Figure 4A). The redox-active Cyt b_6_/f complex was decreased in HL-grown *adg1-1/tpt-2 *compared to wild-type and single mutant plants. Accumulation of Cyt b_559_, an essential structural component of PSII, was unchanged in HL-grown double mutant plants (Figure 4B) despite the overall decrease in PSII core components. Immunoblots of the PsbE protein, which forms the α-subunit of Cyt b_559_, support the spectroscopic determination of Cyt b_559_. The decrease in PsbE was much less pronounced compared for instance to PsbD in HL-grown *adg1-1/tpt-2 *(Figure 4C).

The impaired abundance of PSII core components and the formation of supercomplexes in *adg1-1/tpt-2 *was further supported by separation of thylakoid proteins isolated from HL-grown plants on Blue-Native gels. Most strikingly the abundance of PSII and PSI supercomplexes as well as of PSII monomers was severely diminished in *adg1-1/tpt-2 *compared to the wild type and the single mutants, whereas ATPase and LHCII trimers (which serve as a reference at 110 kDa) were not affected (Figure 4D). Moreover, the high molecular mass band 1 (>1000 kDa) was completely missing in the double mutant. The protein composition of band 1 was determined by LC-MS/MS (Additional File 2). It corresponds well with an earlier report by Peng et al. [27] and suggests that the NDH1-PSI supercomplex is absent in HL-grown *adg1-1/tpt-2.*

### Chl-*a *fluorescence emission at 77 Kelvin reveals a high portion of free, highly fluorescent LHCs uncoupled from their photosystems in *adg1-1/tpt-2*

**Figure 5:**
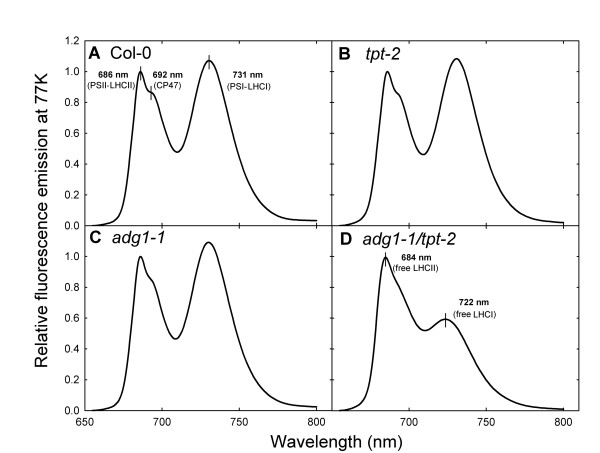
**Fluorescence emission spectra of Chl at 77 K**. Relative fluorescence emission spectra of Chl in isolated thylakoids from Col-0 wild type (A), *tpt-2 *(B), *adg1-1 *(C), and *adg1-1/tpt-2 *(D) determined at 77 K. The spectra were normalized for the peak intensity at 686 nm in case of the wild type and the single mutants, and to the emission maximum of the free LHCII at 684 nm in case of the *adg1-1/tpt-2 *double mutant

The low temperature Chl-*a *fluorescence emission from isolated thylakoids permits an estimate of the functional coupling of LHCs with the reaction centers. While the emission spectra of the wild type and the single mutants were almost identical (Figure 5A to 5C), there were characteristic changes in the spectrum obtained from *adg1-1/tpt-2 *(Figure 5D). The wild type and the single mutants show a maximum emission from PSII-LHCII at 686 nm with a shoulder at 692 nm emitted from CP47. These signals are typical for PSII-LHCII supercomplexes with highly efficient exciton transfer from the outer antennae to the reaction centers. However, in the double mutant the maximum emission is shifted from 686 nm to 684 nm, reflecting a fraction of free LHCII excitonically uncoupled from PSII. Likewise, the emission maximum at 731 nm observed for the wild type and the single mutants is typical for a strong coupling of LHCI to PSI. In the double mutant the emission maximum is shifted from 731 nm to 722 nm, which indicates a high portion of uncoupled LHCI with emission maxima between 705 and 725 nm. These data indicate that a high portion of F_o _in the *adg1-1/tpt-2 *double mutant is emitted from LHCs uncoupled from their photosystems.

### The efficiency of PSI is less affected compared to PSII in HL-grown *adg1-1/tpt-2 *plants

**Figure 6:**
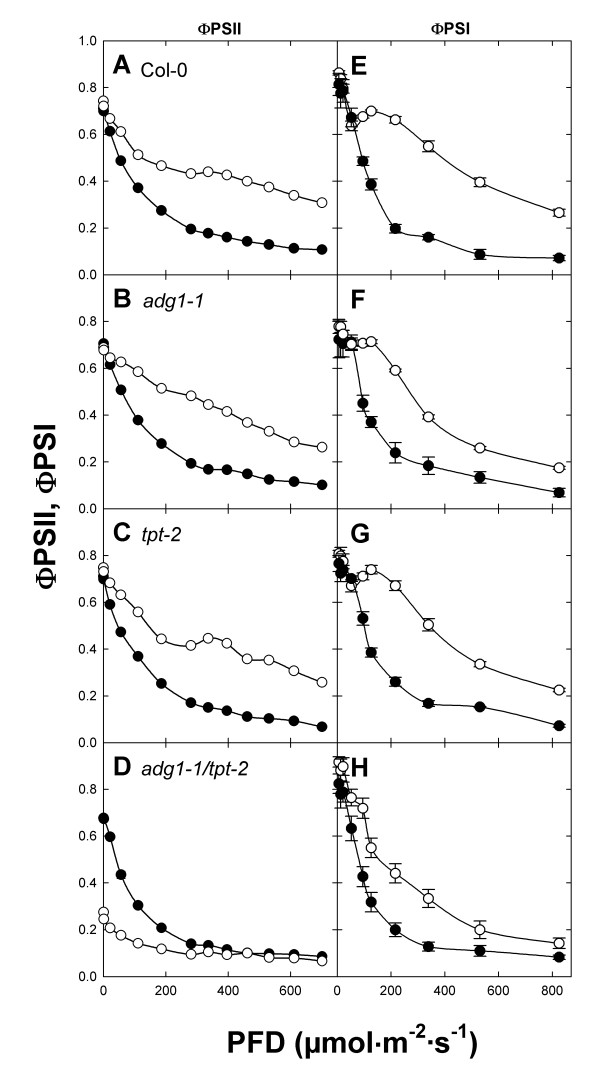
**Light dependencies of ΦPSII and ΦPSI of HL- and LL-grown wild-type and mutant plants**. The response of the quantum efficiencies of PSII (ΦPSII, A-D) or PSI (ΦPSI, E-H) towards increasing light intensities was determined with a DUAL PAM fluorometer for Col-0 wild type (A, E), the *adg1-1 *(B, F) or *tpt-2 *(C, G) single mutants as well as the *adg1-1/tpt-2 *double mutant (D, H) grown in HL (○) or LL (●). The data represent the mean ± SE of 12 independent measurements. Note that for some data the error bars are smaller than the symbol size

Previous determinations of photosynthesis parameters of the *adg1-1/tpt-1 *allele suggested a decrease in the electron transport rate (ETR) through PSII (ETRII) by nearly 90% compared to the wild type or the single mutants [13]. In view of the perturbed stoichiometry of both photosystems as well as a high portion of antennae uncoupled from their reaction centers we compared the quantum efficiencies of PSII and PSI electron transfer (i.e. ΦPSII and ΦPSI) rather than the derived ETRII and ETRI. The light curves of ΦPSII and ΦPSI were similar in LL- and HL-grown wild-type and single mutant plants (Figure 6A to 6C and 6E to 6G), but exhibited characteristic differences in the *adg1-1/tpt-2 *double mutant (Figure 6D and 6H). Up to a PFD of 200 μmol·m^-2^·s^-1 ^the ΦPSII values were lower in HL- compared to LL-grown *adg1-1/tpt-2 *plants (Figure 6D). However, both curves converged at PFDs above 200 μmol·m^-2^·s^-1 ^and ΦPSII had similar values as LL-grown wild-type and single mutant plants. In contrast to the ΦPSII response to increasing PFDs, ΦPSI values were appreciably higher in HL- compared to LL-grown double mutant plants throughout the whole range of PFDs applied (Figure 6H), suggesting that the efficiency of PSI was less affected compared to PSII in HL-grown double mutant plants.

Moreover, donor site limitation of PSI (ΦND, i.e. the limitation of electron transfer from the cytb_6_/f complex to PSI) was increased in HL-grown *adg1-1/tpt-2 *plants, compared to the wild type and the single mutants, while acceptor site limitation (ΦNA, i.e. the limitation of electron transfer from PSI to NADP) was close to zero (Additional File 3). Both parameters remained unaffected between the lines, when the plants were grown in LL. Hence, in HL-grown double mutant plants electron transfer from PSII to PSI is compromised and PSI efficiency is not acceptor site limited.

### Far red and actinic illumination induces non-photochemical quenching of HCF in *adg1-1/tpt-2*

**Figure 7:**
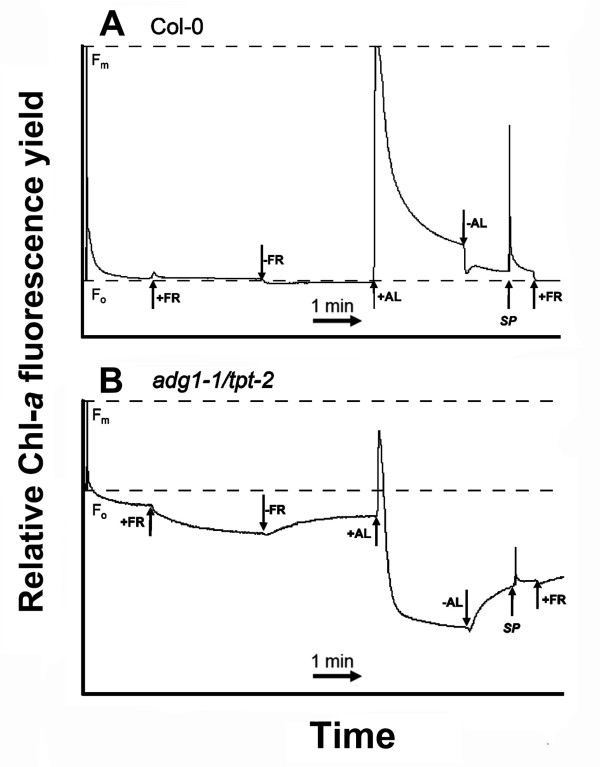
**Time course of Chl-*a *fluorescence yields of wild-type or double mutant plants in response to far red (FR) or actinic light (AL)**. The response of Chl-*a *fluorescence yields of Col-0 wild-type (A) or *adg1-1/tpt-2 *double mutant plants (B) towards FR or AL (at a PFD of 4000 μmol·m^-2^·s^-1^) was determined in time course experiments with a PAM 2100 fluorometer. The plants were dark-adapted for 30 min prior to the experiment. During the course of the experiment a saturation light pulse (SP) was applied after 5 s in order to determine F_o _and F_m_. Where indicated by arrows, FR or AL were either switched on (+) or off (-). SP indicates the application of saturation light pulses

The portion of the LHCs not associated with PSII or PSI core proteins gives rise to the increased Chl-*a *fluorescence yield in dark-adapted HL-grown *adg1-1/tpt-2*. To separate individual components of the increased F_o _in *adg1-1/tpt-2 *the time-course routine outlined in Figure 7A was applied. Upon determination of F_o _and F_m_, plants were illuminated with far red (FR) light to specifically excite PSI (in order to completely oxidize the PSII acceptor site) and subsequently with actinic light (AL). The *adg1-1/tpt-2 *double mutant exhibited a slow decline in the fluorescence yield in the presence of FR until a new minimum level was attained (Figure 7B), whereas F_o _in the wild type remained unaffected by FR (Figure 7A). It is conceivable that this portion of quenching observed in the double mutant is due to re-oxidation of Q_A_. Upon turning FR off the fluorescence signal of *adg1-1/tpt-2 *relaxed again slowly to the original F_o _value observed in dark-adapted plants (Figure 7B). Following the application of strong AL (PFD of approximately 4000 μmol·m^-2^·s^-1^), the fluorescence signal of *adg1-1/tpt-2 *dropped rapidly below F_o _and reached a minimum (F_o_') upon switching off AL (Figure 7B), which was similar to the F_o _observed in wild-type plants (Figure 7A), suggesting that this residual fluorescence was based on LHCs associated with the remaining PSII core components and that the fluorescence of LHCs not associated with PSII was quenched non-photochemically. In contrast to the wild-type, F_o _in *adg1-1/tpt-2 *increased again upon darkening and the subsequent application of a saturating light pulse (SP) caused only a small increase in the fluorescence yield, supporting the idea that non-photochemical quenching (NPQ) of free LCHs is the major cause for the diminished fluorescence yield in AL. However, it is not clear which mechanism is responsible for the decrease in F_o _after the application of FR light to dark-adapted *adg1-1/tpt-2 *leaves.

**Figure 8:**
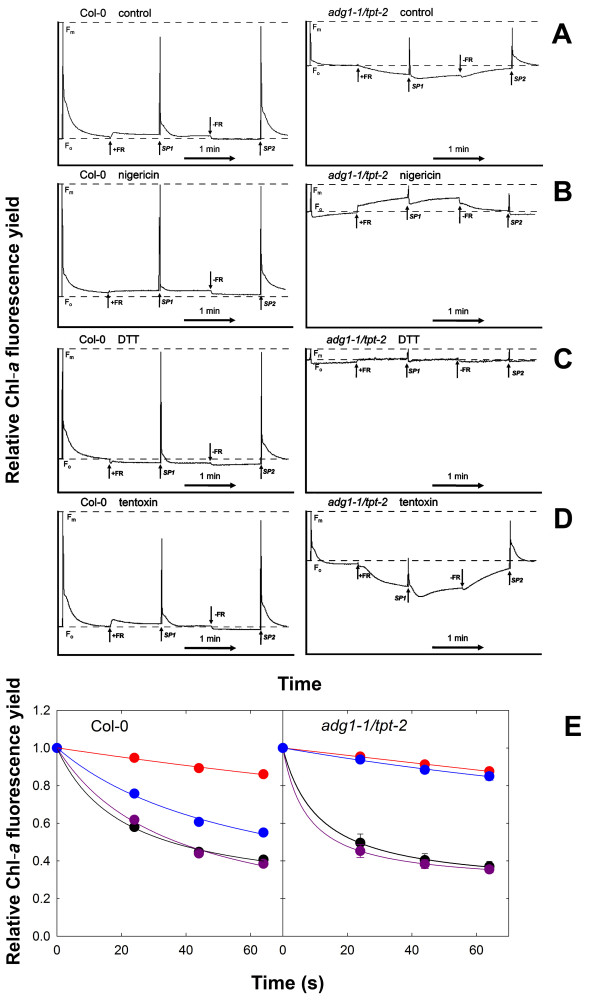
**Effects of nigericin, DTT and tentoxin on the response of Chl-*a *fluorescence to illumination with far red light in dark-adapted wild-type and double mutant plants**. The time course experiments were conducted with a PAM 2100 fluorometer and show the response of Chl-*a *fluorescence yields of Col-0 wild-type or *adg1-1/tpt-2 *double mutant plants toward far red (FR) illumination in the absence (control, A) or presence of nigericin (B), DTT (C) or tentoxin (D). Prior to the measurements detached leaves were incubated either in 0.05% ethanol (control) or inhibitor solutions for 1 h in the dark. During the course of the experiment a saturation light pulse (SP) was applied after 5 s in order to determine F_o _and F_m_. Where indicated by arrows, FR was either switched on (+) or off (-). SP indicates the application of saturation light pulses. (E) Impact of nigericin (red circles), DTT (blue circles) or tentoxin (purple circles) on the decay of maximum Chl-*a *fluorescence yield (F_m_') in leaves of Col-0 and *adg1-1/tpt-2 *plants during illumination with AL at a PDF of 600 μmol·m ^-2^·s ^-1 ^compared to the control (black circles). The data for *adg1-1/tpt-2 *represent the mean ± SE of n = 3 independent experiment

We therefore investigated the mechanistic basis for FR dependent quenching of F_o _in dark-adapted double mutant plants by the application of inhibitors such as nigericin (a protonophor dissipating the transthylakoid proton gradient), dithiothreitol (DTT; a potent inhibitor of the xanthophyll cycle) and tentoxin (a specific inhibitor of thylakoid ATPase). Compared to the control (Figure 8A), the application of nigericin (Figure 8B) and more so of DTT (Figure 8C) to dark-adapted leaves of *adg1-1/tpt-2 *resulted in an increase in F_o _and concomitantly a further decrease in the F_v_/F_m _ratio, whereas in the wild type these parameters remained unaffected by inhibitor treatments. Most strikingly, upon illumination with FR the Chl-*a *fluorescence yield was enhanced rather than diminished in *adg1-1/tpt-2 *and F_m _was identical to the F_m _of dark-adapted leaves both in wild-type and *adg1-1/tpt-2 *plants. It is likely that the decline in F_m _during FR illumination in the control plants (Figure 8A) is due to the establishment of a proton gradient across the thylakoid membrane. Non-photochemical energy quenching (q_E_) of free LHCs induced by this proton gradient is abolished both by nigericin and DTT (Figure 8B and 8C). In contrast, the application of tentoxin resulted in an increase in the F_v_/F_m _ratio of dark-adapted *adg1-1/tpt-2 *leaves and a more pronounced quenching of F_o _upon illumination with FR light (Figure 8D), suggesting that by inhibition of the thylakoid ATPase a steeper pH gradient across the thylakoid membrane was established, which enhanced q_E_. Figure 8E shows the time-dependent response of F_m_' relative to F_m _upon illumination with actinic light (PFD = 600 μmol·m^-2^·s^-1^). The application of nigericin inhibited q_E _similarly in *adg1-1/tpt-2 *and the wild type, whereas the application of DTT had strong inhibitory effects on q_E _in *adg1-1/tpt-2 *and an intermediate effect in the wild type. The presence of tentoxin had no significant impact on q_E _neither in *adg1-1/tpt-2 *nor in the wild type (Figure 8E).

Finally, as for *adg1-1/tpt-1*, deprivation of O_2 _in the dark-adapted state resulted in a further increase in F_o _by approximately 10% in the *adg1-1/tpt-2 *allele, but not in wild-type plants. This surplus of F_o _could rapidly be quenched by FR illumination, indicating a re-oxidation of Q_a_ and the plastoquinone pool via PSI in the absence of O_2 _(Additional File 4).

### The content of soluble sugars, but not the energy state in leaves is severely compromised in *adg1-1/tpt-2 *during the light period

**Figure 9:**
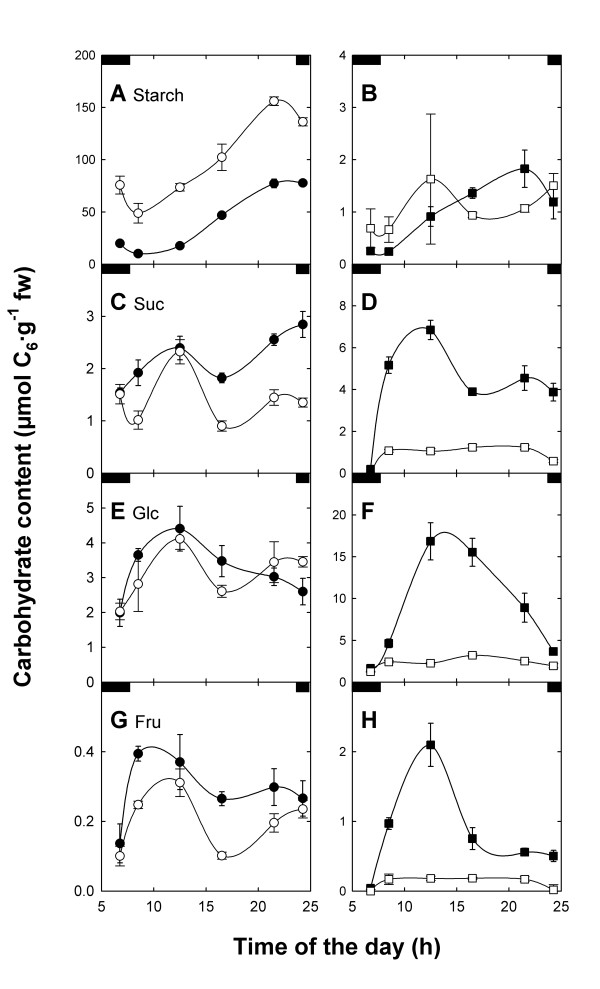
**Diurnal changes in carbohydrate contents in leaves of wild-type and mutant plants.** Contents of starch (A, B), the soluble sugars sucrose (C, D), glucose (E, F) and fructose (G, H) in leaves of Col-0 wild-type (●), the *tpt-2 *(○) and *adg1-1 *single mutants (■) as well as the *adg1-1/tpt-2 *double mutant (□). The data represent the mean ± SE of three independent experiments. Note that the y-axes have been adapted to maximum carbohydrate contents in the individual lines

A restriction on TP export in the light or the deficiency in starch biosynthesis leads either to an accumulation of starch or soluble sugars [13,21]. Hence, a block in both the day- and night-path of photoassimilate export from the chloroplast should result in a severe perturbation of carbohydrate metabolism in leaves of the *adg1-1/tpt-2 *double mutant. Figure 9 shows variations in contents of starch and soluble carbohydrates (sucrose [Suc], glucose [Glc], and fructose [Fru]) during the course of a day in HL-grown *adg1-1/tpt-2 *compared to wild-type and single mutant plants. Apart from the deficiency in producing significant amounts of transitory starch (Figure 9A and 9B), *adg1-1/tpt-2 *is incapable of producing large quantities of soluble carbohydrates during the day (Figure 9D,F and 9H). Therefore the double mutant represents a carbohydrate-starved plant. In contrast, the *adg1-1 *single mutant accumulates soluble sugars, most prominently Glc (Figure 9D). Moreover, small amounts of starch were still detectable both in *adg1-1 *and the double mutant (Figure 9B). Similar to *tpt-1 *[13], the *tpt-2 *allele exhibited increased levels of starch compared to the wild type during the light period (Figure 9A). Furthermore, *tpt-2 *transiently accumulated Suc within the first 4-5 h in the light, followed by a decline in Suc contents until the end of the light period (Figure 9C). The *adg1-1 *single mutant showed a similar transient accumulation of soluble sugars during the course of the day (Figure 9D,F and 9H), reflecting most likely restrictions in feedback regulation mechanism of enzymes involved in sucrose biosynthesis in HL. When the plants were grown in LL, the levels of free sugars between the lines remained largely unaffected (Table 2A).

**Table 2:** Carbohydrate contents of LL-grown plants (A) as well as contents of adenylates in Col-0, *adg1-1, tpt-2 *and *adg1-1/tpt-2 *grown in LL (B) or HL (C)

Plant lines	Starch	Sucrose	Glucose	Fructose
(μmol C6·g^-1 ^fw)				

A Plants grown in LL				

Col-0	4.69 ± 0.59 ^**b**^, ^**d**^	1.71 ± 0.18	1.69 ± 0.48 ^**b**^, ^**d**^	0.53 ± 0.13

*adg1-1*	0.24 ± 0.06 ^**a**^	1.47 ± 0.33	2.46 ± 0.14 ^**a**^, ^**c**^	1.05 ± 0.07

*tpt-2*	3.89 ± 0.47 ^**b**^, ^**d**^	1.10 ± 0.14 ^**d**^	1.04 ± 0.07 ^**b**^, ^**d**^	0.32 ± 0.13

*adg1-1/tpt-2*	0.69 ± 0.54 ^**a**^, ^**c**^	1.36 ± 0.38	1.86 ± 0.16 ^**a**^, ^**c**^	0.68 ± 0.19

**Plant lines**	**ATP**	**ADP**	**AMP**	**EC**

(nmol·g^-1 ^fw)				

B Plants grown in LL				

Col-0	34.0 ± 1.5	14.8 ± 1.1	1.3 ± 0.1	0.83

*adg1-1*	48.4 ± 13.5	21.7 ± 4.9	1.9 ± 0.6	0.82

*tpt-2*	30.1 ± 4.5	12.4 ± 2.0	1.1 ± 0.2	0.83

*adg1-1/tpt-2*	36.3 ± 0.4	16.9 ± 0.9	1.2 ± 0.1	0.82

**Plant lines**	**ATP**	**ADP**	**AMP**	**EC**

(nmol·g^-1 ^fw)				

C Plants grown in HL				

Col-0	159.5 ± 10.5	90.8 ± 5.4	19.9 ± 1.8 ^**d**^	0.76

*adg1-1*	191.9 ± 25.4	90.8 ± 8.6	14.9 ± 2.1	0.80

*tpt-2*	208.4 ± 7.9	106.3 ± 6.3	17.4 ± 3.1 ^**d**^	0.79

*adg1-1/tpt-2*	154.5 ± 11.1	88.0 ± 4.7	9.0 ± 1.2 ^**a**^, ^**c**^	0.79

Leaf samples were taken after 8 h in the light. Metabolism in the leaf samples was quenched immediately in N_2 _during illumination. The data represent the mean ± SE of n = 3 (A) or n = 6 (B and C) measurements. Significant differences in the data pairs were identified by the ANOVA test and further analyzed by the Tukey-Kramer test. The letters as superscripts denote the lines to which a significant difference (P < 0.05) was found with a = Col-0, b = *adg1-1*, c = *tpt-2 *and d = *adg1-1/tpt-2*. The energy charge (EC) was calculated from the relationship EC = ([ATP] + 0.5·[ADP])/([ATP] + [ADP] + [AMP]).

Despite large fluctuations in carbohydrate contents between the plants and a severe growth retardation of HL-grown *adg1-1/tpt-2*, contents of the adenylates ATP, ADP and AMP remained largely unchanged between the different plant lines grown either under LL- or HL-conditions (Table 2B and 2C). Compared to LL-conditions, the average ATP, ADP, and AMP contents were increased 5-, 6-, and 12-fold in all plant lines grown in HL. However, taking the energy charge (EC) as a measure of adenylate pool homeostasis into account, there were only little differences between LL- and HL-grown plants. In all plant lines, the EC of between 0.82 and 0.83 at LL was only slightly higher compared to the EC of between 0.76 and 0.80 at HL. Hence, energy deficiency seems not to be the cause for the observed growth and photosynthesis phenotypes of HL-grown *adg1-1/tpt-2*.

### Sugar feeding rescued the growth and HCF phenotypes of *adg1-1/tpt-2 *plants independently from the induction of GPT2

**Figure 10:**
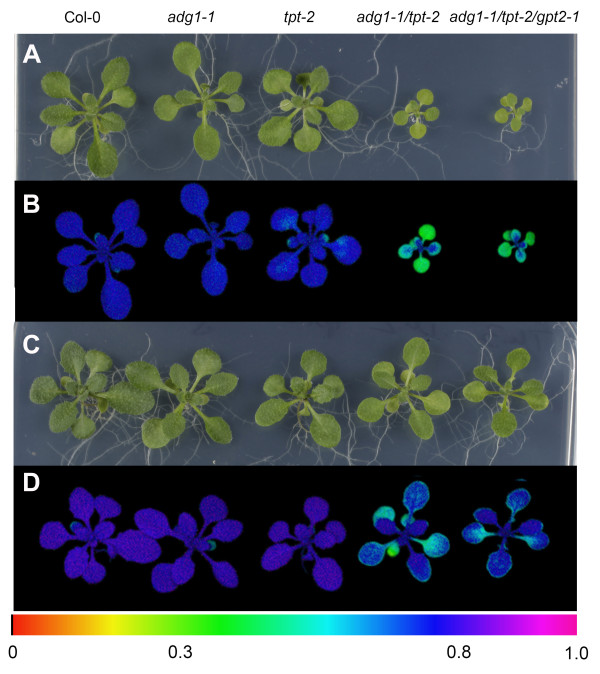
**Phenotypes and Chl-*a *fluorescence images of wild-type and mutant plants grown on MS agar plates in the absence or presence of Suc**. Phenotypic appearance of Col-0, *adg1-1, tpt-2, adg1-1/tpt-2*, and *adg1-1/tpt-2/gpt2-1 *grown under HL-conditions for 4 weeks on MS-agar plates in the absence (A) or presence (C) of 50 mM Suc as well as modulated Chl-*a *fluorescence false color images of the F_v_/F_m _ratio of the same lines in the absence (B) or presence (D) of Suc. The color scale indicates the numeric values of the F_v_/F_m _ratios

Carbohydrate starvation in the *adg1-1/tpt-2 *is correlated with the development of the growth and HCF phenotype under HL conditions. Therefore the impact of external fed sugars on the acclimation response was investigated in HL-grown wild-type and double mutant plants. Both, the growth and HCF phenotypes of *adg1-1/tpt-2 *could be rescued when the plants were grown on MS medium supplemented with 50 mM Suc (Figure 10C and 10D) or 50 mM Glc (not shown) compared to the unfed controls (Figure 10A and 10B). The rescue of the HCF phenotype of *adg1-1/tpt-2 *grown on 50 mM sucrose was reflected in significant increases in the F_v_/F_m _ratio, ΦPSII, Chl contents and the Chl *a/b *ratios (Table 3). The above parameters almost recovered to wild-type level. The recovery of Suc-grown double mutant plants was accompanied by a substantial increase in D2 protein (PsbD) abundance and moderately enhanced phosphorylation states of Lhcb2, and PsbA/PsbD (Additional file 5), indicating that carbohydrate starvation observed in *adg1-1/tpt-2 *might be a main reason for a compromised HL acclimation. In contrast, feeding of Suc in prolonged darkness (i.e. 48 h) failed to rescue the HCF phenotype of *adg1-1/tpt-2 *(not shown).

**Table 3:** Photosynthetic performance as well as pigment contents of Col-0 and *adg1-1/tpt-2 *plants grown on agar plates containing 1/2 strength MS medium (A) or 1/2 MS supplemented with 50 mM Suc (B) under HL-conditions

Plant line	**F**_**v**_**/F**_**m **_**ratio**	ΦPSII	Chl content (mg·g^-1 ^fw)	Chl *a/b *ratio	Carotenoid content (μg·g^-1 ^fw)
A Plants grown on 1/2 MS					

Col-0	0.76 ± 0.01	0.34 ± 0.03	1.43 ± 0.10	3.02 ± 0.14	51.3 ± 4.0

*adg1-1/tpt-2*	0.35 ± 0.10 ^**b**^	0.06 ± 0.05 ^**a**^	0.85 ± 0.14 ^**b**^	2.22 ± 0.08 ^**a**^	34.4 ± 6.0 ^**c**^

B Plants grown on 1/2 MS + 50 mM Suc					

Col-0	0.78 ± 0.01	0.35 ± 0.02	1.64 ± 0.21	2.87 ± 0.06	49.8 ± 7.7

*adg1-1/tpt-2*	0.63 ± 0.06 ^***c***^	0.21 ± 0.04 ^**c, *c***^	1.55 ± 0.21 ^***c***^	2.66 ± 0.03 ***^a^***	59.3 ± 9.0

Photosynthesis parameters were determined with the Imaging-PAM fluorometer using the integrated light source. F_v_/F_m _ratios were determined after the leaves were dark-adapted for at least 15 min. The OPSII values refer to steady state photosynthesis, which was attained 7-10 min after onset of illumination at a PFD of 300 μmol·m^-2^·s^-1^. For pigment and carbohydrate measurements leaves were harvested in the middle of the light period. The data represent the mean ± SE of n = 4 measurements. Significant differences between the data were analyzed according to the Welch test. The letters as superscripts denote ^**a, *a***^*P *< 0.01, ^**b, *b***^*P *< 0.02, ^**c, *c***^*P *< 0.05. Plain letters = comparison of *adg1-1/tpt-2 *with Col-0 at the respective growth conditions, italic letters = comparison of *adg1-1/tpt-2 *plants grown on 50 mM Suc with those grown on 1/2 MS as a control. There were no significant differences found between Col-0 grown either on 1/2 MS or 50 mM Suc.

We have recently demonstrated that the induction of GPT2 at a transcriptional and functional level is correlated with the accumulation of sugars in leaves of mutants defective in starch biosynthesis [21]. Such an additional capacity to transport phosphorylated intermediates across the envelope membrane might compensate for the loss of TPT activity. Indeed, upon Suc feeding a 10-fold increase in *GPT2 *transcript abundance (determined by qRT-PCR) in *adg1-1/tpt-2 *compared to only 1.4-fold in the wild type was detected. Hence, recovery of the double mutant's phenotype in the presence of Suc could be entirely based on the induction of GPT2. To test this assumption, we generated a homozygous *adg1-1/tpt-2/gpt2-1 *triple mutant, which was phenotypically indistinguishable from the *adg1-1/tpt-2 *double mutant (Figure 10A and 10B). Strikingly, the triple mutant could also be rescued by Suc feeding (Figure 10C and 10D). This observation indicates that GPT2 does obviously not play a key role in the acclimation response to HL of *adg1-1/tpt-2 *upon Suc feeding.

### A general block in the day- and night path of carbon export from the chloroplast leads to HCF and growth phenotypes similar to *adg1-1/tpt-2*

The characteristic growth and HCF phenotypes are not restricted to *adg1-1/tpt-2*, but are also evident in double mutants impaired in the TPT in combination with other steps of starch biosynthesis such as phosphoglucose isomerase (*pgi1-1*) or phosphoglucomutase (*pgm1*), i.e. the *pgi1-1/tpt-2 *and *tpt-2/pgm1 *double mutants (Additional File 6A). Like *adg1-1/tpt-2*, these double mutants lack any pronounced phenotype when grown in LL (Additional File 6A). Strikingly, the high-starch mutant *mex1-2/tpt-2 *impaired in both the maltose transporter and the TPT also exhibits a phenotype similar to *adg1-1/tpt-2 *(Additional File 6B). However, this phenotypic similarity is not based on a high starch level per se, as the *sex1-3/tpt-2 *double mutant (Additional File 6C), impaired in the GWD protein and the TPT, does not show a pronounced HCF phenotype. A comparison of the Chl-*a *fluorescence characteristics between the lines revealed that, apart from *sex1-3/tpt-2*, all other double mutants showed an increased F_o_, and a similar response towards AL illumination, i.e. a fluorescence quenching below F_o _followed by a slow recovery in the dark (not shown). However, the quenching of F_o _by FR illumination was absent both in *sex1-3/tpt-2 *and *mex1-2/tpt-2.*

**Figure 11:**
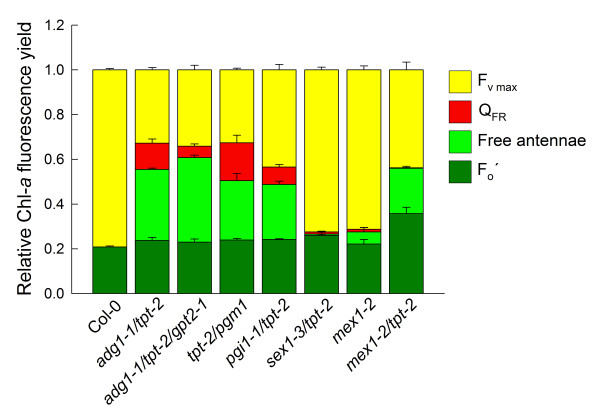
**Summarizing analysis of Chl-*a *fluorescence yields of dark-adapted wild-type and mutant plants**. The analysis is based on similar time course experiments as shown in Figure 7. The individual bars indicate fluorescence emitted from LCHs associated with PSII (dark green bars; F_o_, i.e. after application of AL at a PFD of 4000 μmol·m^-2^·s^-1^), whereas (light green bars) and (red bars) indicate the relative fluorescence yield emitted from free antennae, which either can (light green bars) or cannot (red bars) be partially quenched by FR illumination (Q_FR_). The yellow bars represent the maximum F_v _emitted from PSII reaction centers. The data were normalized for F_m _(= 1) and represent the mean ± SE of n = 3 measurements

The relative contribution of individual fluorescence classes to the total fluorescence yield, i.e. F_o _emitted from LHCs coupled to or uncoupled from their photosystems as well as the portion of F_o _quenching by FR illumination and the maximum variable fluorescence (F_m_) is summarized in Figure 11 and is based on Chl-*a *fluorescence time course experiments similar to those shown in Figure 7. In *adg1-1/tpt-2, adg1-1/tpt-2/gpt2-1, tpt-2/pgm1 *and *mex1-2/tpt-2*, F_m _was severely diminished compared to the wild type or the *sex1-3/tpt-2 *double mutant, whereas the fluorescence of free antennae was enhanced indicating that a severe restriction in leaf carbohydrate metabolism promotes a massive loss of PSII integrity under HL-conditions. Following application of a very high intensity of AL (PFD = 4000 μmol·m^-2^·s^-1^) the F_o_' (i.e. the fluorescence emission from antennae associated with their phostosystems) was, apart from *mex1-2/tpt-2*, very similar in all lines compared to the wild type. In the latter double mutant F_o_' was 1.75-fold higher than in the wild-type. Moreover, in all starch-free or low-starch double mutants a substantial part of the HCF could be quenched by FR light, most likely by thermal dissipation of excitation energy. In contrast, FR light had no effect on the HCF of *mex1-2/tpt-2.*

## Discussion

In this report we have studied the acclimation response of plants with a strongly perturbed leaf carbohydrate metabolism. The major outcome of our investigations can be summarized as follows: (i) double mutants impaired in the day- and night path of photoassimilate export are still viable, (ii) the growth and HCF phenotypes develop only when the mutant plants were grown in HL and were virtually absent in LL-grown plants. (iii) Growth in HL resulted in severe changes in the composition of thylakoid proteins involved in photosynthetic electron transport characterized most prominently by a decrease in the plastome-encoded core components of both photosystems leading to increased contents of uncoupled, highly fluorescent nuclear encoded PSII and PSI antennae. Loss of both PSII and PSI reaction centers led to diminished Chl contents and a drop of the Chl *a/b *ratio in *adg1-1/tpt-2*. (iv) Feeding of soluble sugars such as Suc or Glc rescued the growth and HCF phenotypes of the *adg1-1/tpt-2 *double mutant and the *adg1-1/tpt-2/gpt2-1 *triple mutant indicating that either sugar signaling is involved in the altered HL acclimation in carbohydrate-starved plants or alternative NADPH and ATP consuming metabolic pathways are enhanced. Moreover, GPT2 is not a key player in the sugar-triggered rescue of the mutant's phenotype.

### Residual TPT activity or the induction of *GPT2 *is not responsible for survival of double mutants impaired in the day- and night path of photoassimilate export

A block in starch biosynthesis and the day path of photoassimilate export results in growth retardation and a HCF phenotype of the plants (i.e. *adg1-1/tpt-2, tpt-2/pgm1, pgi1-1/tpt-2*). It has previously been proposed that residual TPT activity (which can be as high as 16% compared to the wild type) in the background of *adg1-1/tpt-1 *[21] might permit survival of this double mutant allele [13,20]. With the aid of the newly established *adg1-1/tpt-2 *double mutant, which completely lacks *TPT*-specific transcripts and TPT transport activity, we could clearly demonstrate that the TPT is not required for survival. It is likely that the induction of other phosphate translocators can compensate for the absence of the TPT. For instance, one of the two *A. thaliana GPT *genes (i.e. *GPT2) *is induced in leaves of mutants defective in starch biosynthesis (such as *adg1-1, pgm1 *and *pgi1-2*) as well as in the *adg1-1/tpt-1 *double mutant [21]. It has been proposed that GPT2 is an essential component of acclimation to HL in *A. thaliana *[28]. In wild-type plants both *GPT *genes are either not (*GPT2*) or only weakly (*GPT1*) expressed in photoautotrophic tissues [29]. As GPT proteins are capable of transporting TPs besides of Glc6P [30], the presence of a functional GPT in chloroplasts would relief the constraints of photoassimilate export by the TPT. With the aid of the *adg1-1/tpt-2/gpt2-1 *triple mutant we could demonstrate that GPT2 is not involved in the survival of the double mutant. However, it is conceivable that a small portion of TPs can be exported via the xylulose 5-P (Xu5P)/phosphate translocator (XPT, At5g17630; [31]), which is, according to the electronic fluorescent protein browser, expressed in autotrophic and heterotrophic tissues of *A. thaliana (*http://www.bar.utoronto.ca/efp/cgi-bin/efpWeb.cgi; [32]). Besides its specific transport capacity for Xu5P, the XPT accepts TP, but not 3-PGA as a substrate with a K_m _value in a physiological range (i.e. 0.4 mM; [31]). There is, however, no indication that the *XPT *gene is upregulated in the double mutant background (unpublished data). Future experiments have to clarify whether *adg1-1/tpt-2/gpt2-1/xpt *quadruple mutants are still viable.

### The HCF phenotype of *adg1-1/tpt-2 *is caused by antennae uncoupled from diminished PSII and PSI core proteins

Mutants impaired in the day and night path of carbon export from the chloroplasts (i.e. *adg1-1/tpt-2, tpt-2/pgm1, pgi1-1/tpt-2*) develop a HCF phenotype when grown in HL. The high F_o _is accompanied by a significant quenching of the fluorescence yield below F_o _during illumination with AL. These pattern of fluorescence changes is reminiscent of PSII mutants [33], for instance defective in PSII stability [34] or its assembly, such as *lpa1 *[35], *lpa2 *[36], or most recently in the thylakoid protein PAM68 [37]. Based on the fluorescence emission spectra at 77 K and immunoblot analyses, we could demonstrate that the major part of HCF in HL-grown *adg1-1/tpt-2 *plants derives from changes in the composition or contents of thylakoid proteins The most prominently decreased proteins were the plastome-encoded core proteins of both photosystems (D1, D2, CP43, CP47, P700 apoprotein A2), with the exception of the Cyt b_559 _subunit PsbE and the β-subunit of thylakoid ATPase (AtpB), whereas the contents of their LHCs or other nuclear-encoded proteins such as PsbO or PetC (Rieske) remained rather unchanged or were even increased (FNR). Free antennae are highly fluorescent [38,39] and give rise to the observed HCF phenotype.

Due to the inevitable formation of reactive oxygen species, PSII is constantly damaged, particularly under HL-conditions [40]. D1 and D2 exhibit a high protein turnover, as they are subject to PSII photoinhibition (reviewed by [41-43]). In *adg1-1/tpt-2 *double mutant plants exposed to HL-conditions, the decomposition rate of PSII core proteins presumably exceeds the rate of their *de novo *synthesis. Moreover, PSI abundance in *adg1-1/tpt-2 *declined in line with PSII. The basis of the reduced PSI content is still unknown. Normally, PSI content remains unaffected in response to PSII photoinhibition [44,45].

Strikingly, the abundance of Cyt b_559_, a plastome-encoded constituent of PSII remained high despite of a severe decline in D1 or D2. Normally, the content of Cyt b_559 _is strictly correlated with the other PSII core proteins in chloroplasts, and free Cyt b_559 _does not accumulate. In case of PSII photoinhibition, Cyt b_559 _is degraded in parallel with the rest of the PSII reaction center [46]. However, free Cyt b_559 _has for instance been observed in etioplasts prior to PSII assembly during de-etiolation [47,48] or in a screen for HCF mutants, particularly those affected in PSII [33]. It has recently been proposed that Cyt b_559 _acts as a plastoquinole oxidase [49]. It remains to be determined if free Cyt b_559 _also has a photoprotective function. Strikingly, a deficiency in Cyt b_559 _rather than an increased Cyt b_559 _abundance leads to a similar HCF phenotype in a tobacco mutant, which has been interpreted as an over-reduction of the plastoquinone pool [49,50]. Likewise the HCF phenotype of tobacco plants kept and illuminated for a prolonged time in CO_2_-free air [51] or of *Beta vulgaris *plants suffering from iron deficiency [52] has been attributed to a reduced PSII acceptor site.

We have recently proposed that the HCF phenotype observed in the *adg1-1/tpt-1 *double mutant might also be connected to an increased reduction state of Q_A _and the plastoquinone pool (PQ) in the dark [20]. This assumption appeared reasonable considering that deprivation of O_2 _or the inhibition of chloroplast alternative oxidase (PTOX), involved in chlororespiration, by octyl gallate (OG) further increased the F_o _in the double mutant, but not in the wild type. This surplus of Chl-*a *fluorescence yield could readily be quenched by the application of FR light, indicating that indeed electrons residing at the PSII acceptor site were removed efficiently via PSI. A similar 10% rise in HCF as a response to O_2 _deprivation and a rapid drop of the additional fluorescence yield by FR illumination has also been observed for the *adg1-1/tpt-2 *double mutant allele (see Additional File 4). If this surplus of HCF was based on chlororespiration [53], NDH1 would probably not take part in electron transfer as the NDH1-PSI supercomplex is missing in *adg1-1/tpt-2 *(Figure 4D, Additional File 2). However, the major portion of the HCF in both the *adg1-1/tpt-1 and adg1-1/tpt-2 *alleles remained unaffected by FR illumination (compare Figure 7B, and [20]) and is emitted from LHCs decoupled from their photosystems.

### Thermal dissipation of excitation energy leads to quenching of HCF below F_o_

Non-photochemical quenching (NPQ) protects the photosystems (in particular PSII) from overexcitation by increased thermal dissipation in the PSII antennae [54,55], which requires the acidification of the thylakoid lumen, the protonation of LHCs [56]) as well as PsbS [57] and an operational xanthophyll cycle, e.g. [58]. These mechanisms were proposed to change the conformation of PSII antenna proteins and PsbS [59-61], which leads to a decline in the lifetime of excited Chl molecules within the antennae ([62,63]) and results in a higher probability of thermal dissipation rather than excitation transfer to the reaction centers.

Here we provide evidence that the fluorescence of free LHCs can be quenched by thermal dissipation both in actinic light and even in FR light. The kinetics of slow fluorescence decay (in a minutes scale) during FR illumination and its slow recovery in the dark suggest conformational changes of the LHC proteins. Time constants in the range of 45 s, as has been observed in *adg1-1/tpt-2 *(compare Figure 7B), would be typical for such conformational changes [64]. Moreover, the application of the protonophore nigericin and the xanthophylls cycle inhibitor DTT dramatically diminished NPQ in the double mutant background. Strikingly, HCF quenching upon application of FR light was completely abolished in the presence of nigericin and DTT and showed a trend of an increase in the presence of tentoxin, a specific inhibitor of thylakoid ATPase (Compare Figure 8). The application of FR light to dark-adapted wild-type leaves had only little effect on Chl-*a *fluorescence. From these observations, we conclude that FR illumination is capable of generating a proton gradient, potentially due to cyclic electron transport (CET) around PSI, which leads to some NPQ of free antennae.

Interestingly, with the exception of the *mex1-2/tpt-2 *double mutant, the F_o_' value upon illumination with high PFDs of AL was almost identical in all the mutant lines tested with a comparable HCF phenotype. Hence, this remaining portion of fluorescence yield might reflect the emission from LHCs functionally coupled with their photosystems. In contrast, the mild HCF phenotype in the *mex1-2/tpt-2 *double mutant might be based on mechanical lesions of chloroplasts as has recently been shown for the *mex1 *single and *dpe1/mex1 *[6] or *tpt-2/mex1 *double mutants [7].

### Is the diminished phosphorylation state of thylakoid proteins responsible for grana hyperstacking in the *adg1-1/tpt *double mutants?

Plants can adapt to fluctuating light by state transitions [65], which dominate at low light intensities and involve the reversible phosphorylation/dephosphorylation of PSII antennae by a specific kinase (STN7; [66,67]) and phosphatase (TAP38, [68,69]). This leads to decoupling of the phosphorylated PSII antennae from the core of PSII and the lateral movement to and association with PSI. By this mechanism the distribution of excitation energy between the photosystems can be adapted to the requirements of the plants [70]. Furthermore the STN8 kinase is responsible for the phosphorylation of PSII core proteins such as D1 [66,71] and hence involved in the PSII repair cycle [43,72,73] after photoinhibition. Here we could show that the phosphorylation states of Lhcb2 as well as of PsbC, CaS, and PsbA/PsbD, were severely diminished in HL-grown *adg1-1/tpt-2 *plants compared to the wild type. Whilst the decline in the phosphorylation states of PsbC and PsbA/PsbD correlates well with a decrease in their total contents, the abundance of Lhcb2 remained unaffected in *adg1-1/tpt-2 *and would hence result in a high portion of non-phosphorylated PSII antennae. The decrease in phosphorylated PSII core protein content combined with a diminished Lhcb2 phosphorylation would be consistent with an increased grana stacking in *adg1-1/tpt-2*. A modified grana stacking has also been observed in *stn8 *and *stn7 *mutants [74,75]. Moreover, like *adg1-1/tpt-1 *and *adg1-1/tpt-2 *alleles, plants grown under LL-conditions [76-78] or light qualities that favor the excitation of PSI [79] exhibit an increased grana stacking. It remains to be solved whether a more oxidized PSII acceptor site in the double mutant triggers the degree of grana stacking.

### External supply of sugars rescues the growth and HCF phenotypes of *adg1-1/tpt-2*

Externally supplied Suc or Glc could rescue the growth phenotype of *adg1-1/tpt-2*. This is not surprising as the double mutant is neither capable of accumulating starch nor large amounts of soluble sugars. Hence feeding of sugars to carbohydrate-starved plants ought to provide sufficient carbon precursors for vegetative growth. However, the rescue of the HCF phenotype of the double mutant cannot be easily explained by a supply of carbon skeletons as an additional energy source for ATP biosynthesis. The energy charge (EC) of LL- and HL-grown mutant and wild-type plants remained largely unchanged in the light, suggesting that a limitation of ATP (e.g. for the PSII repair cycle) is not a major reason for the HCF phenotype or the observed retardation in growth. Strikingly, in the absence of light, feeding of sugars was not effective in alleviating the HCF phenotype of *adg1-1/tpt-2 *(not shown). It is conceivable that the absence or very low levels of soluble sugars blocks signaling pathways required for a proper acclimation response. Hence sugar signaling, i.e. *via *hexokinase [80] might be directly or indirectly involved in this response, whereas the involvement of GPT2 in the sugar-dependent rescue of the *adg1-1/tpt-2 *growth and HCF phenotypes can be ruled out as a similar rescue occurred also in the absence of GPT2 in the a*dg1-1/tpt-2/gpt2-1 *triple mutant. Moreover, application of sugars or a changing light environment alters the metabolic signature in the mesophyll, which might itself be recognized as a signal [81,82]. Apart from its role as signaling molecule the presence of Glc (or Suc) might enhance metabolic sequences within the chloroplast that act as NADPH and ATP sinks in the light such as biosynthesis of the photosynthetic machinery or de novo fatty acid biosynthesis and thereby relieve photoinhibition.

## Conclusions

Here we demonstrate that carbohydrate limitation compromises acclimation to HL in *A. thaliana*. It is conceivable that the strong relationship between the chloroplast and nucleus with respect to a co-ordinated expression of photosynthesis genes is modified. This is shown by the severe repression of plastome-encoded subunits of the photosynthetic complexes and the unaltered accumulation of nuclear-encoded components such as LHCs and the subunits of the oxygen evolving complex. The generation and/or transmission of chloroplast signals to the nucleo/cytosolic system might be strongly altered during HL-exposure of *adg1-1/tpt-2 *compared to the wild type or the single mutants. Therefore, the *adg1-1/tpt-2 *double mutant may serve as an excellent tool to study principles of retrograde signaling essential for the coordinated expression of nuclear- and plastome-encoded photosynthesis genes in the future. This aspect will be addressed by transcriptomic and metabolomic approaches. Moreover, it ought to be considered that transcription and translation of plastome-encoded genes ([40,83,84] as well as the assembly of the photosystems within the thylakoid membrane are partially controlled by nuclear-encoded factors [85,86].

## Methods

### Plant material and growth conditions

Seeds of *A. thaliana *ecotypes Ws-2, Col-0, and Ler were obtained from the Nottingham Arabidopsis Stock Centre (NASC). In addition, the following mutant lines defective in the genes indicated were used: *gpt2-1 *(At1g61800; [29]), *adg1-1 *(At5g48300; [16]), *sex1-3 *(At1g10760; [24]), *pgm1 *(At5g51820; [22[), *pgi1-1 *(At4g24620; [23]), and *mex1-2 *(At5g17520; [5]).

Plants were germinated and grown on soil for approximately 4 weeks in a growth cabinet (Percival, CLF Plant Climatics GmBH, Wertingen, Germany, model AR-36 L3/HIL) at a light/dark cycle of 16 h/8 h, a day/night temperature of 22°C/18°C and at relative humidity of 40%. Each of the three levels of the growth cabinet was equipped with 14 fluorescence tubes (Osram L18W/840), which could be dimmed to the desired PFD. For growth in HL or LL, the photosynthetic active PFD was adjusted to 300 μmol·m^-2^·s^-1 ^or 30 μmol·m^-2^·s^-1 ^at leaf level and controlled once a week by a LI-COR LI 250 light meter. Feeding experiments with exogenous carbohydrates were performed after seeds were germinated and grown on sterile 1/2 Murashige-Skoog (MS) agar or on 1/2 MS agar supplemented with 50 mM Suc or 50 mM Glc for 4 weeks under HL conditions as described above.

### Isolation of the *tpt-2 *mutant allele and generation of double mutants

The mutant line N573707 (SALK_073707.54.25.x, Col-0 background) was screened via standard PCR on genomic DNA using g_tpt-2 primers in combination with T-DNA border primers (Additional File 7). The resulting PCR product was sequenced and the T-DNA insertion in the *TPT *gene (At5g46110) 8 bp downstream of the start ATG was verified. The isolated homozygous *tpt-2 *mutant plants were smaller in size and were hence backcrossed to Col-0 wild type. Only those plants of the F2 population were further propagated that carried the *tpt-2 *allele homozygously and lacked any growth retardation compared to Col-0. The *tpt-2 *mutant line was crossed to *adg1-1, pgm1, pgi1-1, sex1-3 *and *mex1-2*. Immature flowers of homozygous single mutants were emasculated and manually cross pollinated. The crosses of *tpt-2 × adg1-1, pgi1-1, sex1-3*, and *mex1-2*, and were performed using the *adg1-1, pgi1-1*, and *mex1-2 *as the female parent. For the cross of *pgm1 *× *tpt-2 *the *tpt-2 *plant was used as the female parent. Triple mutants lacking TPT, ADG and GPT2 were generated by crossing the double mutants *adg1-1/tpt-2 *to *adg1-1/gpt2-1 *[21]. The progenies of the crosses were screened either for starch-free or starch excess phenotypes by iodine staining at the end of a light period or at the end of a prolonged dark period and for T-DNA insertions. For primers used see Additional File 7.

### Protein isolation and immunoblot analyses

Samples for protein isolation and subsequent immunoblot analyses were taken directly in the Percival growth chamber under LL- and HL-conditions after 4 h of illumination. Total proteins of leaves were extracted as described in [87]. Of the protein extract 10 μg was separated by discontinuous 12.5% SDS-polyacrylamide gelelectrophoresis (PAGE) and electroblotted on polyvinylidene fluoride (PVDF) membranes (Bio-Rad). The membranes were incubated overnight in a casein containing blocking solution and probed with primary antibodies against photosynthesis associated proteins supplied by Agrisera (Vännäs, Sweden). Following incubation with the secondary antibody, i.e. horseradish peroxidase conjugate (Sigma, St. Louis, *Missouri)*, the proteins were detected by chemoluminescence in the presence of SuperSignal West substrate (Thermo Fisher Scientific) by exposure to a film (*CL-XPosure*, Thermo Scientific). For the detection of phosphorlylated proteins a specific phospho-threonine antibody (Cell signaling, Beverly, Massachusetts) was used as the primary antibody. As casein is highly phosphorylated, it was replaced by BSA in the blocking solution.

### Isolation of thylakoid membranes and blue-native-PAGE

Native isolation of thylakoid membranes was performed according to [88] with 10 mM NaF added to the extraction media. Membrane proteins were solubilized with 2% n-dodecyl-β-D-maltoside in solubilization buffer (50 mM Bis-Tris [pH 7.0], 750 mM 6-aminocaproicacid; 5 mM EDTA [pH 7.0]; 50 mM NaCl). The samples were separated on commercially available continuous 3-12% native Bis-Tris acrylamide gels (Invitrogen) as described in [89] and stained with colloidal Coomassie G [90]. For the identification of protein supercomplexes, proteins separated by BN-PAGE in a first dimension were further separated by SDS-PAGE in the second dimension according to [41].

### Mass spectroscopic analysis of proteins

For the further separation and identification of protein supercomplexes on BN-gels, individual protein bands were excised, subjected to a tryptic digestion and the peptides separated by nano-liquid chromatography (Proxeon, Odense, Dänemark), ionized by electrospray ionisation (ESI) and detected and fragmented by tandem MS *(HCT Ultra ETD II*, Bruker Daltonik, Bremen). The obtained mass and fragment spectra were compared to the NCBInr database (*A. thaliana*) with the help of Mascot (*Matrixscience*; [91]) at a significance level of (*P *< 0.05) and a mass tolerance of ± 0.3 Da.

### RNA extraction and qRT-PCR

RNA was extracted according to [92]. After treatment with DNA-free DNase (Ambion), oligo(dt)-primed cDNA of total RNA was synthesized using the Bioscript reverse transcriptase (Bioline). *GPT2 *transcript abundance was analyzed by quantitative RT-PCR with the SYBR Green PCR Master Mix (Applied Biosystems) in combination with the 7300 Sequence Detection System (Applied Biosystems). Relative transcript amounts were quantified with the AAC_t _method [93]. As a control ubiquitin C (UBC, At5g25760) was used according to [94]. For gene specific primers see Additional File 7.

### Metabolite determination

Starch and soluble sugars were isolated from frozen leaf material according to [16] and determined with a coupled enzymatic assay [95] in a Spectrafluor Plus plate reader (TECAN, Switzerland) in the absorbance mode. For the determination of metabolites with a high turnover such as nucleotides, care was taken that metabolism in the leaf samples was quenched immediately in liquid N_2 _during illumination with LL or HL. Nucleotides were extracted from around 100 mg of leave material using perchloric acid. After derivatization with chloracetaldehyd the remaining etheno-adensosine nucleotides were separated and quantified by HPLC (Dionex Ultimate LC 3000) as described by [96].

### Determination of pigment and protein contents

Plant material was ground in liquid nitrogen and pigments associated with photosynthesis were extracted in 100% acetone. Chl content as well as Chl *a/b *ratios were assayed in 80% acetone and calculated as described in [97]. Total carotenoids were determined at a wavelength of 480 nm and the contents calculated using the equation, Car = E_480 _+ 0.144·E_663 _- 0.638·E_645_. For total protein content measurement, plant material was extracted in 50 mM Hepes-NaOH (pH 7.0) in the presence of 0.1% Triton-X-100. Protein contents were determined as described in [98].

### Determination of TPT transport activity

TPT transport activity was determined after reconstitution of membrane proteins extracted from *A. thaliana *leaves into artificial liposomes, according to the method described by [99] using radiolabeled ^32^P_i _as a counter exchange substrate.

### *In vivo *determination of PSII and PSI performance

Performance of PSII was determined by Chl-*a *fluorescence measurements with the pulse amplitude modulation fluorometers Imaging-PAM or PAM-2100 (Walz, Effeltrich, Germany). The individual fluorescence parameters determined by the 'saturation-pulse-method' are defined according to [100]. For inhibitor studies excised leaves were incubated for 1 h in aqueous solutions containing 6.5 μM tentoxin, 100 μM nigericin [101] or 3 mM DTT [102] in 0.01% (v/v) ethanol. As a control leaves were incubated in a 0.01% (v/v) ethanol solution.

Performance of PSI was determined from light-induced absorption changes at a wavelength of 830 nm minus 870 nm [103] with the Dual-PAM (Walz, Effeltrich, Germany). PSI can be completely oxidized by the application of FR light followed by a saturating light pulse (SP), yielding the P_m _value, which is analogous to F_m _at PSII. During illumination with actinic light (AL) the reduction state of PSI varies depending on the PFD as well as on acceptor- or donor site limitations. The portion of still oxidizable PSI in the light (P_m_') can be determined by the application of SPs during AL illumination. The performance of PSI is derived from the three quantum efficiencies ΦNA (acceptor site limitation), ΦND (donor site limitation), and ΦPSI (electron transport). The sum of these three parameters equals one. The quantum efficiencies of electron transport is defined as ΦPSI = 1 - ΦND - ΦNA.

### Thylakoid membrane isolation, quantification of photosynthetic complexes and Chl-*a *fluorescence emission analysis at 77 K

Thylakoid membranes were isolated according to [104]. The contents of Cyt b_559_, which can be usually used to quantify PSII, and of the Cyt b_6_/f complex were determined from difference absorption signals of Cyt b_559_, f and b_6_. Thylakoids equivalent to 50 μg Chl·ml^-1 ^were destacked in a low salt medium, to improve the optical properties of the sample [105]. All cytochromes were oxidized by the addition of 1 mM potassium ferricyanide (+III), and subsequently reduced by addition of 10 mM sodium ascorbate and dithionite, resulting in the reduction of Cyt f and the high-potential form of Cyt b_559 _(ascorbate-ferricyanide difference spectrum) and of Cyt b_6 _and the low-potential form of Cyt b_559_, respectively. At each redox potential, absorption spectra were measured between 575 and 540 nm wavelength with a V-550 spectrophotometer (Jasco GmbH, Groß-Umstadt, Germany) equipped with a head-on photomultiplier. The spectral bandwidth was 1 nm and the scanning speed was 100 nm·min ^-1^. Difference absorption spectra were deconvoluted using reference spectra and difference extinction coefficients as in [105]. PSII contents were calculated from the sum of the high-and low-potential difference absorption signals of Cyt b_559 _[106]. The content of redox-active PSI was determined from light-induced difference absorption changes of P_70_0, the PSI reaction center Chl-*a *dimer. Isolated thylakoids equivalent to 50 μg Chl·ml ^-1 ^were solubilized with 0.2% (w/v) n-dodecyl-β-D-maltoside in the presence of 100 μM paraquat as electron acceptor and of 10 mM sodium ascorbate as electron donor. P_700 _was oxidized by the application of a saturating light pulse (2000 μmol photons·m^-2^·s^-1 ^red light, 200 ms duration). Measurements were done using the Dual-PAM instrument (Walz, Effeltrich, Germany) in its PC-P_700 _version [107].

Chl-*a *fluorescence emission spectra at 77 K were measured on isolated thylakoids equivalent to 10 μg Chl·ml^-1 ^using a F-6500 fluorometer (Jasco GmbH, Groß-Umstadt, Germany). The samples were excited at 430 nm wavelength (10 nm bandwidth). The emission spectra between 655 and 800 nm were recorded with a bandwidth of 1 nm and a scanning speed of 200 nm. Ten spectra were averaged, to increase the signal to noise ratio, and corrected for the instrumental response of the photomultiplier.

### Transmission electron microscopy

Pieces of fully developed leaves were quickly fixed in 2% glutaraldehyde in 50 mM phosphate buffer (pH 7.2). Water was substituted with acetone and subsequently acetone was substituted with Spurr-media [108]. During this process samples were post-fixed and contrasted with 2% osmium tetroxide and 1% uranyl acetate. Polymerized Spurr embedded samples were cut with glass knifes on an ultramicrotome *MT-600*, (RMC, Tuson, USA) and mounted on coated copper grids. Ultrathin sections were post stained with lead citrate and uranyl acetate and viewed in an EM10 electron microscope (Carl Zeis, Jena, Germany).

### Statistical evaluation of experimental data

The data are expressed as mean values ± standard error (SE) of the indicated number of independent measurements. Significant differences between two data sets were analyzed using the Welch-test, which allows for unequal relative errors between two groups of measurements assuming that a Gauss distribution is applicable [109]. Significant differences between more than two data sets were analyzed using ANOVA implemented in Excel. Data sets which passed the ANOVA as significant different were submitted to the post hoc Tukey-Kramer test, which allows the comparison of unequal sample sizes and finds which mean values are significantly different from one another [110] The Tukey-Kramer test was performed with xlstat implemented in Excel. For data plotting, SigmaPlot8.0 for Windows (SPSS Inc.) was used.

## Authors' contributions

JS contributed to the major part of this work such as PS measurements (PAM 2100, DUAL-PAM, Imaging PAM), immunoblots, Blue-Native gelelectrophoresis, carbohydrate determinations, preparations of samples for TEM as well as the molecular characterization of mutant and double mutant plant. MAS carried out Chl fluorescence measurements with isolated thylakoids at 77 K as well as spectroscopic determinations of thylakoid electron transport components. SK performed the determination of adenylates. SG was involved in the TEM analyses. AS originally isolated the *tpt-2 *mutant and established a homozygous line. TK performed the ANOVA analysis and together with DL helped with the immunoblot analyses of thylakoid proteins. KB crossed a variety of mutant plants and established double and triple mutant lines. UIF and REH conceived the project and participated in the design of the study and its coordination. All authors read and approved the final manuscript.

## Supplementary Material

Additional file 1 **Distribution of 'grana stack number classes' in LL- and HL- grown wild-type and double mutant plants.** The number of stacks of 33 to 105 individual grana was determined on TEM images of two to three chloroplasts per line and grouped into classed between 2 and 17 stacks per granum**.** The number of each class was expressed as percentage of the total number of grana counted. The distribution of grana stack numbers was calculated from (A) 105, (B) 77, (C) 75, and (D) 33 individual grana of LL- and HL-grown Col-0 (A, B) or *adg1-1/tpt-2 *(C, D). Click here for file

Additional file 2 **Protein composition of the >1000 kDa supercomplex.** Thylakoid proteins were isolated from wild-type plants by Blue-Native PAGE. The respective protein band was cut out and further analyzed by LC/MS2. The ion score represents -10·Log(P), where P is the probability that an observed match is a random event. Ion scores >38 indicate either identity or extensive homology (*P *< 0.05). Proteins marked by an asterisk indicate putative new subunits of the NDH Supercomplex (compare [27]). Click here for file

Additional file 3 **Light dependency of acceptor- and donor site limitation of PSI determined with HL- and LL-grown wild-type and mutant plants.** Quantum efficiencies of acceptor (blue circles, ΦNA) or donor site (red circles, ΦND) limitation of HL- and LL-grown Col-0 wild-type (A, E), *adg1-1 *(B, F) and *tpt-2 *(C, G) single mutant as well as the *adg1-1/tpt-2 *(D, H) double mutant plants obtained from light saturation curves. The data represent the mean ± SE of 12 independent measurements. Click here for file

Additional file 4 **Effects of O_2 _deprivation on Chl-*a *fluorescence.** Chl-*a *fluorescence traces of HL-grown Col-0 and *adg1-1/tpt-2 *double mutant plants flushed either with air, i.e. in the presence of 21% O_2 _(A, B), or with N_2_, i.e. in the absence of O_2 _(C, D) in a closed Perspex chamber. Where indicated by arrows, FR illumination was either switched on (+FR) or off (-FR). SP indicates the application of saturated light pulsed at a duration of 0.8 s. Click here for file

Additional file 5 **Immunoblots of thylakoid proteins of sucrose-fed wild-type and *adg1-1/tpt-2 *plants compared to the unfed controls.** Immunoblots of photosynthesis associated proteins after separation of 10 μg total protein isolated from HL-grown Col-0 and *adg1-1/tpt-2 *double mutant on SDS-PAGE. Plants were grown either in the absence (MS) or presence of 50 mM Suc. P*-Threonin indicates signals obtained following incubation of the blots with a phospho-threonin antibody. The numbers indicate signals from PsbC (1), CaS (2), PsbA/PsbD (3), and LhcbII (4). Click here for file

Additional file 6 **Phenotypes and Chl-*a *fluorescence images of wild-type and mutant plants.** Description of data: Phenotypic appearance as well as modulated Chl-*a *fluorescence false color images of the F_v_/F_m _ratio of the same lines grown in HL or LL for 4 weeks. (A) *pgi1-1, pgm1, pgi1-/tpt-2, tpt-2/pgm1*, (B) *mex1-2, mex1-2/tpt-2*, (C) *sex1-3, and sex1-3/tpt-2*. The color scale indicates the numeric values of the F_v_/F_m _ratios. Click here for file

Additional file 7 **Oligonucleotide primers.** Primers used for the identification of T-DNA mutants by PCR or transcript amounts by RT-PCR or qRT-PCR (RL). Click here for file
