# Supplementary material for: Defects in leaf carbohydrate metabolism compromise acclimation to high light and lead to a high chlorophyll fluorescence phenotype in Arabidopsis thaliana
Source: BMC Plant Biol. 2012 Jan 16;12:8. doi: 10.1186/1471-2229-12-8 (PMC3353854; doi:10.1186/1471-2229-12-8)
Supplement: Additional file 1 — Distribution of 'grana stack number classes' in LL- and HL- grown wild-type and double mutant plants. The number of stacks of 33 to 105 individual grana was determined on TEM images of two to three chloroplasts per line and grouped into classed between 2 and 17 stacks per granum. The number of each class was expressed as percentage of the total number of grana counted. The distribution of grana stack numbers was calculated from (A) 105, (B) 77, (C) 75, and (D) 33 individual grana of LL- and HL-grown Col-0 (A, B) or adg1-1/tpt-2 (C, D). [file 1471-2229-12-8-S1.PDF]

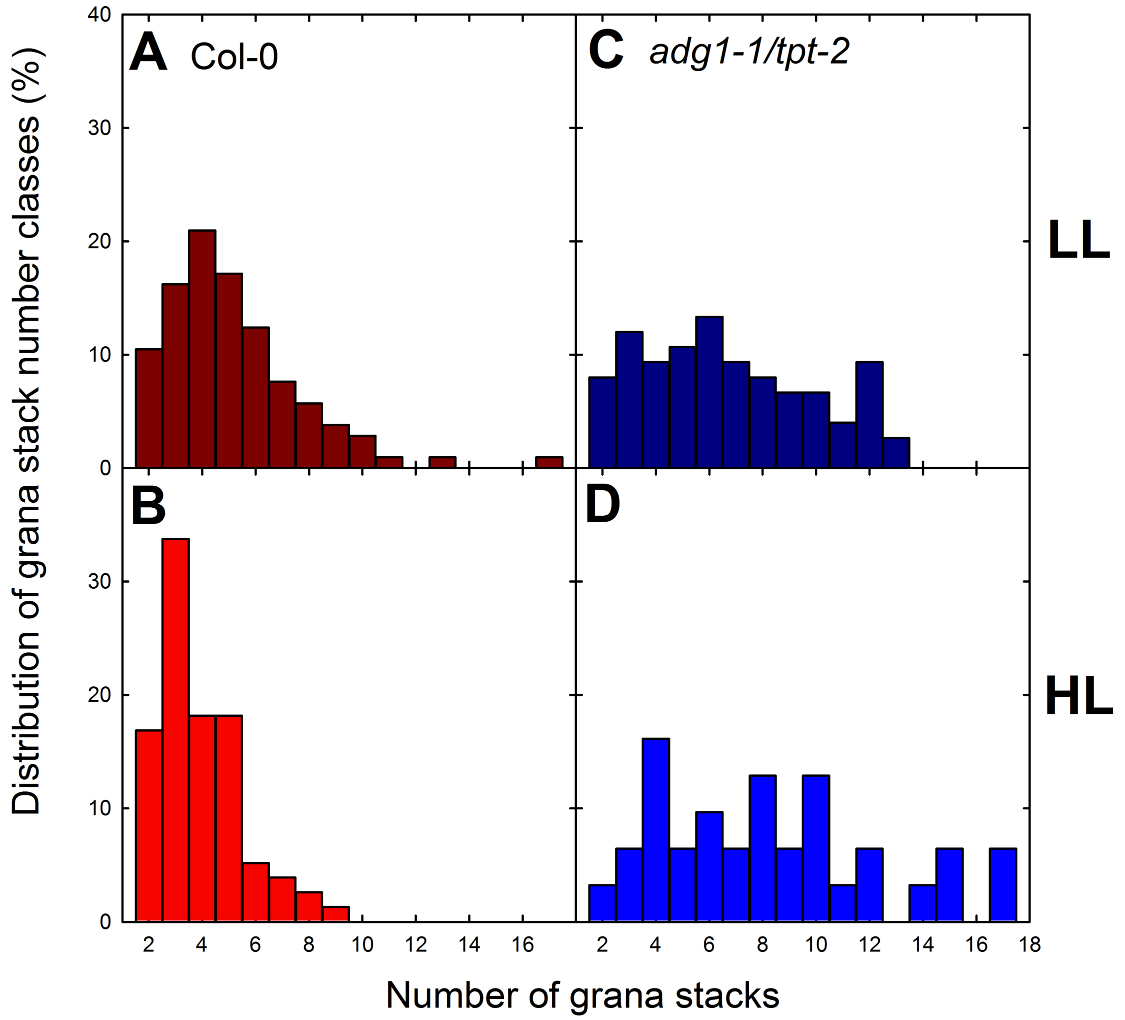

Additional File 1 – Distribution of `grana stack number classes´ in LL- and HL-grown wild-type and double mutant plants

The number of stacks of 33 to 105 individual grana was determined on TEM images of two to three chloroplasts per line and grouped into classed between 2 and 17 stacks per granum. The number of each class was expressed as percentage of the total number of grana counted. The distribution of grana stack numbers was calculated from (A) 105, (B) 77, (C) 75, and (D) 33 individual grana of LL- and HL-grown Col-0 (A, B) or *adg1-1/tpt-2* (C, D).
