# Supplementary material for: Defects in leaf carbohydrate metabolism compromise acclimation to high light and lead to a high chlorophyll fluorescence phenotype in Arabidopsis thaliana
Source: BMC Plant Biol. 2012 Jan 16;12:8. doi: 10.1186/1471-2229-12-8 (PMC3353854; doi:10.1186/1471-2229-12-8)
Supplement: Additional file 2 — Protein composition of the >1000 kDa supercomplex. Thylakoid proteins were isolated from wild-type plants by Blue-Native PAGE. The respective protein band was cut out and further analyzed by LC/MS2. The ion score represents -10·Log(P), where P is the probability that an observed match is a random event. Ion scores >38 indicate either identity or extensive homology (P < 0.05). Proteins marked by an asterisk indicate putative new subunits of the NDH Supercomplex (compare [27]). [file 1471-2229-12-8-S2.PDF]

Additional File 2 - Protein composition of the >1000 kDa supercomplex

Thylakoid proteins were isolated from wild-type plants by Blue-Native PAGE. The respective protein band was cut out and further analyzed by LC/MS2. The ion score represents -10·Log(P), where P is the probability that an observed match is a random event. Ion scores >38 indicate either identity or extensive homology (P<0.05). Proteins marked by an asterisk indicate putative new subunits of the NDH Supercomplex (compare [27]).

	Protein	AGI code	MM[1](kDa)	Number of peptides	Coverage (%)	Score
NDH Complex	NAD7*	AtMg00510	45.5	7	15.3	331.8
	NDF1*	At1g15980	51.0	8	18.7	392.4
	NDF2*	At1g64770	38.0	5	18.1	221.4
	NDF6*	At1g18730	12.8	1	9.3	54.4
	NDH-A	AtCg01100	39.9	2	5	92.3
	NDH-B.1	AtCg00890	57.1	1	1.8	47.5
	NDH-D	AtCg01050	56.9	2	4.2	97.2
	NDH-E	AtCg01070	11.3	1	8.9	70.7
	NDH-F	AtCg01010	30.5	3	8.5	61.4
	NDH-I	AtCg01090	20.1	5	25.6	222.2
	NDH-K	AtCg00430	25.3	5	18.7	203.4
	NDH-M	At4g37925	24.8	1	9.2	52.6
	NDH-N	At5g58260	23.4	4	21.5	173.7
	NDH-O	At1g74880	17.6	1	5.7	42.1
	NHD-J	AtCg00420	18.5	5	30.4	302.5
	PPL2*	At2g39470	26.9	4	17.6	170.1
	PQL1*	At1g14150	22.1	5	21.6	224.9
	PQL2*	At3g01440	24.8	4	14.5	135.4
PSI Complex	Lhca1	At3g54890	26.0	1	5.4	47.1
	Lhca3	At1g61520	29.1	6	27.1	296.2
	Lhca4	At3g47470	16.7	2	23	97.5
	Lhca5*	At1g45474	27.8	3	13.7	157.8
	Lhca6	At1g19150.1	30.0	3	9.2	59.3
	PsaA	AtCg00350	83.2	4	5.3	191.1
	PsaB	AtCg00340	82.4	5	6.4	285.4
	PsaD-1/-2	At1g03130/At4g02770	22.3	6	22.1	208.9
	PsaE1	At4g28750	11.7	2	19.1	147.7
	PsaF	At1g31330	24.2	6	27.6	375.8
	PsaG	At1g55670	17.1	3	10.6	91.3
	PsaH-1/-2	At1g52230/At3g16140	15.3	2	15.2	95.7
	PsaL	At4g12800	23.1	3	16.4	171.8
	PsaN	At5g64040	18.4	1	5.8	54.2
PSII Complex	Lhcb1	At1g29910/At1g29930/At2g34420	25.0	3	12.9	139.6
	Lhcb 4	At5g01530	31.2	2	9.7	112.8
	Lhcb 5	At4g10340	30.1	3	12.5	176.7
	Lhcb 6	At1g15820	27.5	2	7	60.4
	PsbA	AtCg00020	38.9	2	6.5	134.2
	PsbB	AtCg00680	56.0	5	12.4	321.5
	PsbC	AtCg00280	51.8	3	6.8	161.3
	PsbD	AtCg00270	39.5	4	10.8	199.1
	PsbE	AtCg00580	9.4	2	22.9	131.3
	PsbL	AtCg00560	4.5	1	36.8	76.8
	PsbO-1/-2	At5g66570/At3g50820	35.1	1	2.4	40.5
Others	DNAJ HSP	At5g21430	24.4	4	27.1	232.5
	MYB-like TF	At3g04450	48.9	2	4.1	42.8
