# Supplementary material for: Defects in leaf carbohydrate metabolism compromise acclimation to high light and lead to a high chlorophyll fluorescence phenotype in Arabidopsis thaliana
Source: BMC Plant Biol. 2012 Jan 16;12:8. doi: 10.1186/1471-2229-12-8 (PMC3353854; doi:10.1186/1471-2229-12-8)
Supplement: Additional file 3 — Light dependency of acceptor- and donor site limitation of PSI determined with HL- and LL-grown wild-type and mutant plants. Quantum efficiencies of acceptor (blue circles, ΦNA) or donor site (red circles, ΦND) limitation of HL- and LL-grown Col-0 wild-type (A, E), adg1-1 (B, F) and tpt-2 (C, G) single mutant as well as the adg1-1/tpt-2 (D, H) double mutant plants obtained from light saturation curves. The data represent the mean ± SE of 12 independent measurements. [file 1471-2229-12-8-S3.PDF]

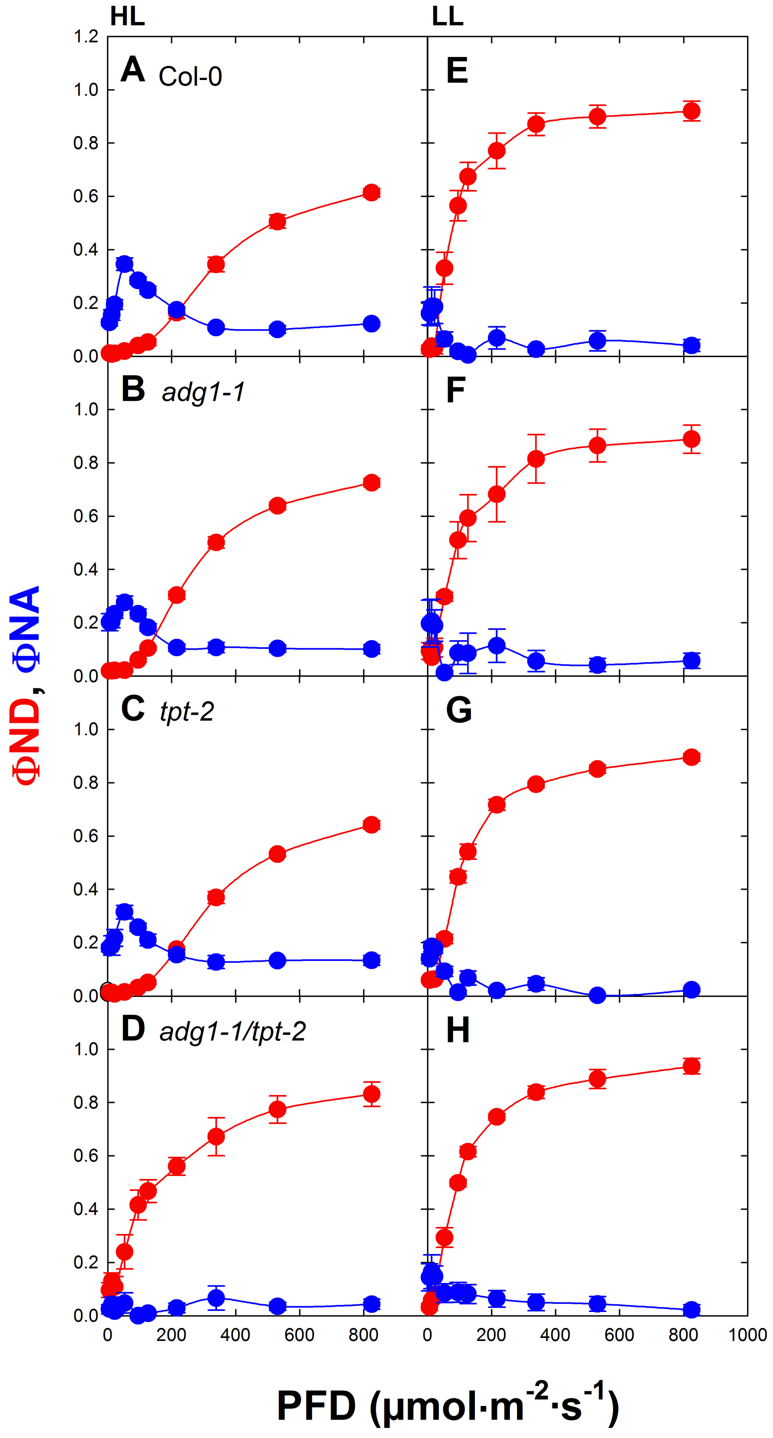

Additional File 3 - Light dependency of acceptor- and donor site limitation of PSI determined with HL- and LL-grown wild-type and mutant plants

Quantum efficiencies of acceptor (●, ΦNA) or donor site (●, ΦND) limitation of HL- and LL-grown Col-0 wild-type (A, E), *adg1-1* (B, F) and *tpt-2* (C, G) single mutant as well as the *adg1-1/tpt-2* (D, H) double mutant plants obtained from light saturation curves. The data represent the mean ± SE of 12 independent measurements.
