# Supplementary material for: Defects in leaf carbohydrate metabolism compromise acclimation to high light and lead to a high chlorophyll fluorescence phenotype in Arabidopsis thaliana
Source: BMC Plant Biol. 2012 Jan 16;12:8. doi: 10.1186/1471-2229-12-8 (PMC3353854; doi:10.1186/1471-2229-12-8)
Supplement: Additional file 4 — Effects of O2 deprivation on Chl-a fluorescence. Chl-a fluorescence traces of HL-grown Col-0 and adg1-1/tpt-2 double mutant plants flushed either with air, i.e. in the presence of 21% O2 (A, B), or with N2, i.e. in the absence of O2 (C, D) in a closed Perspex chamber. Where indicated by arrows, FR illumination was either switched on (+FR) or off (-FR). SP indicates the application of saturated light pulsed at a duration of 0.8 s. [file 1471-2229-12-8-S4.PDF]

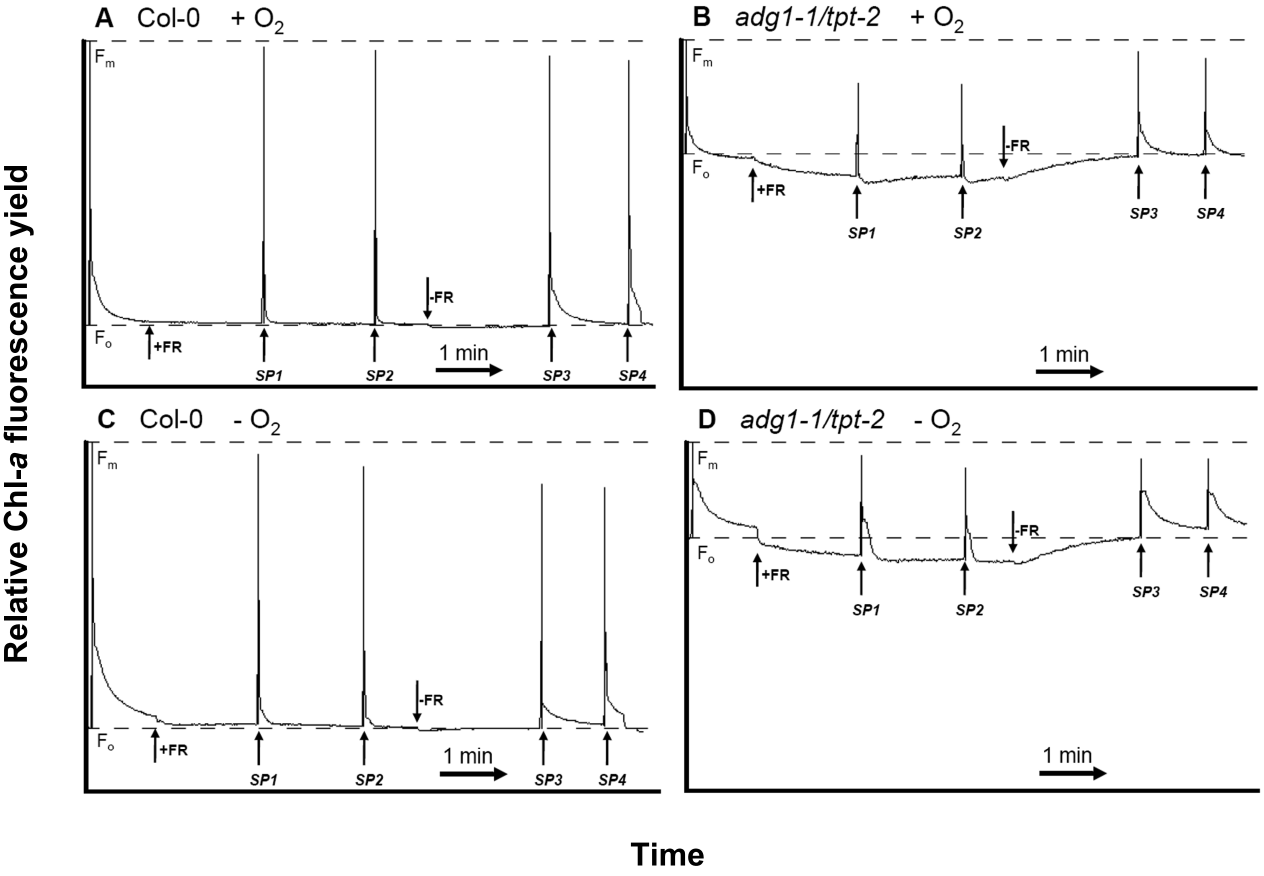

Additional File 4. - Effects of O_2 deprivation on Chl-*a* fluorescence

Chl-*a* fluorescence traces of HL-grown Col-0 and *adg1-1/tpt-2* double mutant plants flushed either with air, i.e. in the presence of 21% O_2 (A, B), or with N_2, i.e. in the absence of O_2 (C, D) in a closed Perspex chamber. Where indicated by arrows, FR illumination was either switched on (+FR) or off (-FR). SP indicates the application of saturated light pulsed at a duration of 0.8 s.
