# Supplementary material for: Defects in leaf carbohydrate metabolism compromise acclimation to high light and lead to a high chlorophyll fluorescence phenotype in Arabidopsis thaliana
Source: BMC Plant Biol. 2012 Jan 16;12:8. doi: 10.1186/1471-2229-12-8 (PMC3353854; doi:10.1186/1471-2229-12-8)
Supplement: Additional file 5 — Immunoblots of thylakoid proteins of sucrose-fed wild-type and adg1-1/tpt-2 plants compared to the unfed controls. Immunoblots of photosynthesis associated proteins after separation of 10 μg total protein isolated from HL-grown Col-0 and adg1-1/tpt-2 double mutant on SDS-PAGE. Plants were grown either in the absence (MS) or presence of 50 mM Suc. P*-Threonin indicates signals obtained following incubation of the blots with a phospho-threonin antibody. The numbers indicate signals from PsbC (1), CaS (2), PsbA/PsbD (3), and LhcbII (4). [file 1471-2229-12-8-S5.PDF]

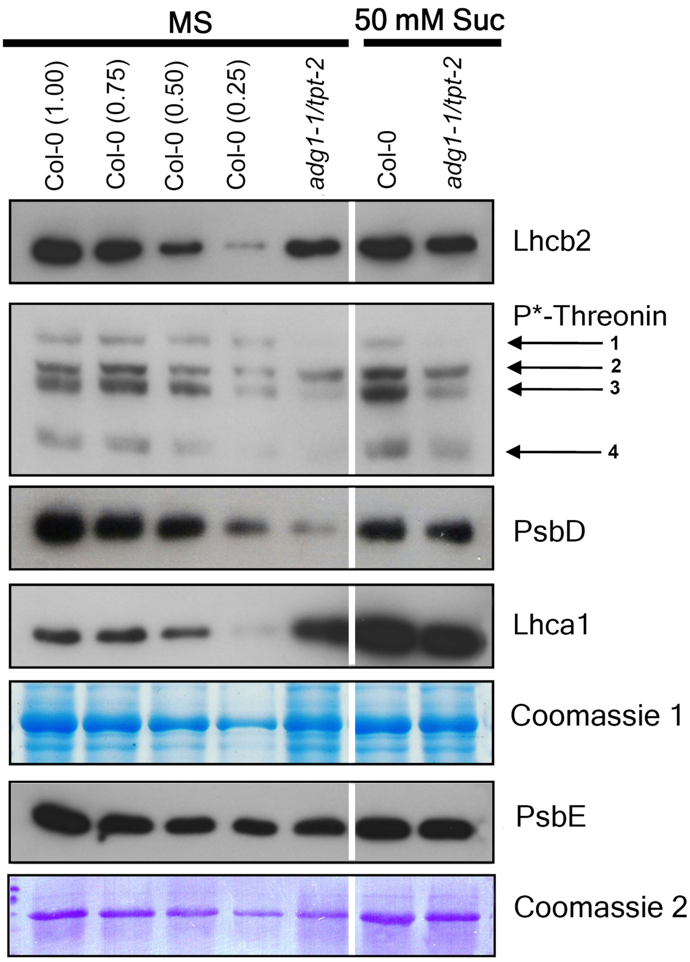

Additional File 5 - Immunoblots of thylakoid proteins of sucrose-fed wild-type and *adg1-1/tpt-2* plants compared to the unfed controls

Immunoblots of photosynthesis associated proteins after separation of 10 µg total protein isolated from HL-grown Col-0 and *adg1-1/tpt-2* double mutant on SDS-PAGE. Plants were grown either in the absence (MS) or presence of 50 mM Suc. P*-Threonin indicates signals obtained following incubation of the blots with a phospho-threonin antibody. The numbers indicate signals from PsbC (1), CaS (2), PsbA/PsbD (3), and LhcbII (4).
