# Supplementary material for: Defects in leaf carbohydrate metabolism compromise acclimation to high light and lead to a high chlorophyll fluorescence phenotype in Arabidopsis thaliana
Source: BMC Plant Biol. 2012 Jan 16;12:8. doi: 10.1186/1471-2229-12-8 (PMC3353854; doi:10.1186/1471-2229-12-8)
Supplement: Additional file 6 — Phenotypes and Chl-a fluorescence images of wild-type and mutant plants. Description of data: Phenotypic appearance as well as modulated Chl-a fluorescence false color images of the Fv/Fm ratio of the same lines grown in HL or LL for 4 weeks. (A) pgi1-1, pgm1, pgi1-/tpt-2, tpt-2/pgm1, (B) mex1-2, mex1-2/tpt-2, (C) sex1-3, and sex1-3/tpt-2. The color scale indicates the numeric values of the Fv/Fm ratios. [file 1471-2229-12-8-S6.PDF]

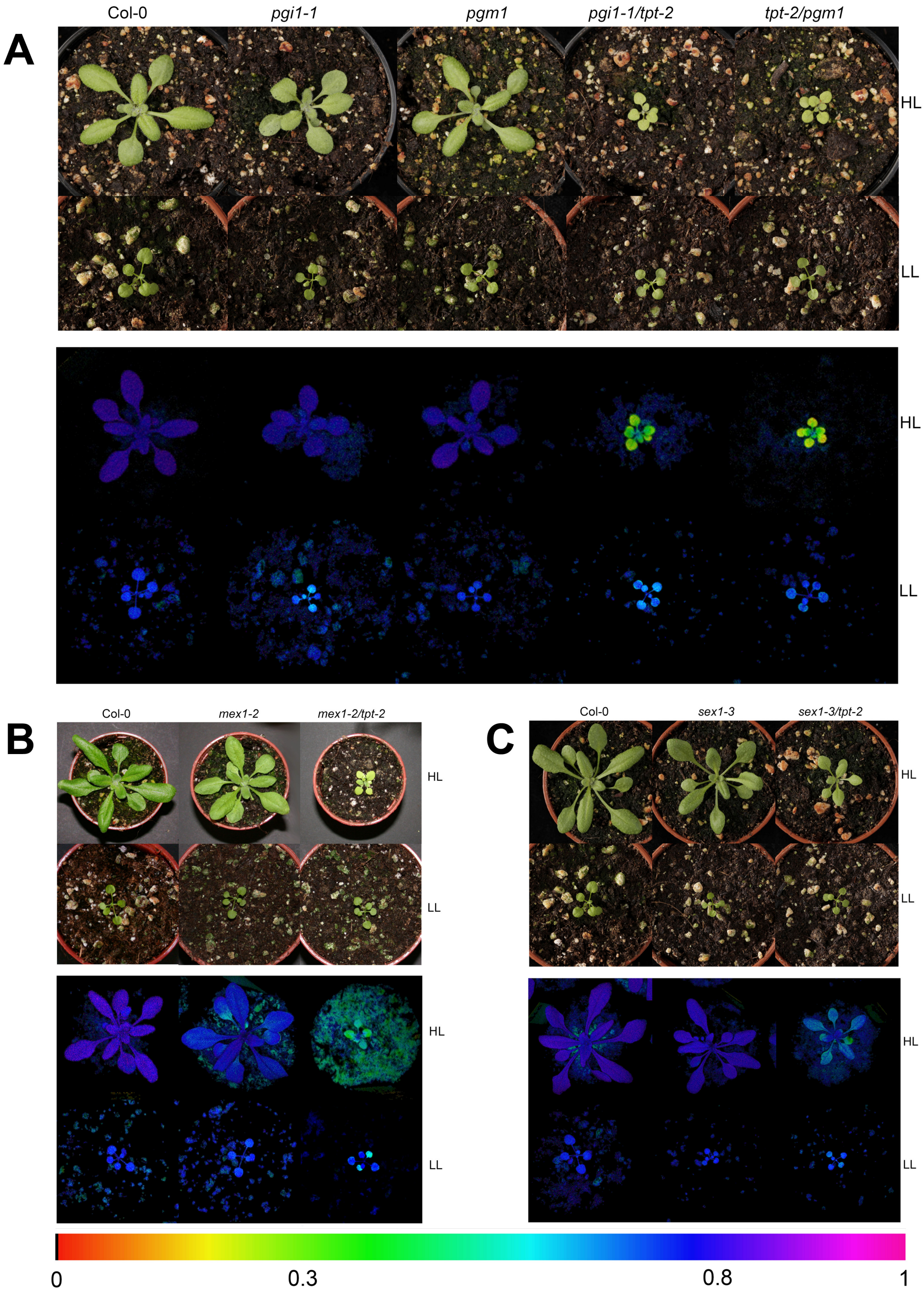

Additional File 6 - Phenotypes and Chl-*a* fluorescence images of wild-type and mutant plants

Phenotypic appearance as well as modulated Chl-*a* fluorescence false color images of the F_v/F_m ratio of the same lines grown in HL or LL for four weeks. (A) *pgi1-1, pgm1, pgi1-/tpt-2*, *tpt-2/pgm1*, (B) *mex1-2, mex1-2/tpt-2,* (C) *sex1-3, and sex1-3/tpt-2*. The color scale indicates the numeric values of the F_v/F_m ratios.
