# Supplementary material for: Defects in leaf carbohydrate metabolism compromise acclimation to high light and lead to a high chlorophyll fluorescence phenotype in Arabidopsis thaliana
Source: BMC Plant Biol. 2012 Jan 16;12:8. doi: 10.1186/1471-2229-12-8 (PMC3353854; doi:10.1186/1471-2229-12-8)
Supplement: Additional file 7 — Oligonucleotide primers. Primers used for the identification of T-DNA mutants by PCR or transcript amounts by RT-PCR or qRT-PCR (RL). [file 1471-2229-12-8-S7.PDF]

Additional File 7 - Oligonucleotide primers

Primers used for the identification of T-DNA mutants by PCR or transcript amounts by RT-PCR or qRT-PCR (RL).

Name	Sequenz 5'-3'
g_tpt-2 fwd	GTA ACT TAC GAG TAA ACT GGC TAC
g_tpt-2 rev	TGA CTA GCC ATG GAT ACT TGG CGA GGA
TPT fwd	TGC TCT CAC TAT CAA AAC TCT GAA
TPT rev	TGA CAG ACT GCG ACT GGT ATC AA
2 TPT fwd	CGA TCA ACG GAG GAG AGA AA
2 TPT rev	GAT ACT TGG CGA GGA ATC CA
g_gpt-2 fwd	GTC GGA CCA AAC TTT GTC TGG T
g_gpt-2 rev	GGT CTG ATC AAG AAA TGA CAC TGA
Salk_LB	GTC CGC AAT GTG TTA TTA AGT TGT C
RL_gpt-2 fwd	TGC CCT CGG TGC TGC CAT TG
RL_gpt-2 rev	CCT CAC TGC TTC GCC TGT GAG T
RL_UBC fwd	CTG CGA CTC AGG GAA TCT TCT AA
RL_UBC rev	TTG TGC CAT TGA ATT GAA CCC
